# Immunogenicity, Safety, and Protective Efficacy of Mucosal Vaccines Against Respiratory Infectious Diseases: A Systematic Review and Meta-Analysis

**DOI:** 10.3390/vaccines13080825

**Published:** 2025-07-31

**Authors:** Jiaqi Chen, Weitong Lin, Chaokai Yang, Wenqi Lin, Xinghui Cheng, Haoyuan He, Xinhua Li, Jingyou Yu

**Affiliations:** 1 Nanshan School, Guangzhou Medical University, Guangzhou 510180, China; drchenjiaqi@gmail.com (J.C.); 2021111263@stu.gzhmu.edu.cn (W.L.); 2022111357@stu.gzhmu.edu.cn (H.H.); dogenotfoundwastaken@gmail.com (X.L.); 2 Guangzhou National Laboratory, Bio-Island, Guangzhou 510005, China; 3 The Third School of Clinical Medicine, Guangzhou Medical University, Guangzhou 510180, China; 2022111364@stu.gzhmu.edu.cn (C.Y.); 2023111388@stu.gzhmu.edu.cn (X.C.); 4 The Second School of Clinical Medicine, Guangzhou Medical University, Guangzhou 510180, China; 2023111282@stu.gzhmu.edu.cn; 5 State Key Laboratory of Respiratory Disease, The First Affiliated Hospital of Guangzhou Medical University, Guangzhou 510182, China

**Keywords:** mucosal vaccine, respiratory infectious diseases, immunogenicity, vaccine efficacy, safety, meta-analysis

## Abstract

**Background/Objectives:** Mucosal vaccines, delivered intranasally or via inhalation, are being studied for respiratory infectious diseases like COVID-19 and influenza. These vaccines aim to provide non-invasive administration and strong immune responses at infection sites, making them a promising area of research. This systematic review and meta-analysis assessed their immunogenicity, safety, and protective efficacy. **Methods**: The study design was a systematic review and meta-analysis, searching PubMed and Cochrane databases up to 30 May 2025. Inclusion criteria followed the PICOS framework, focusing on mucosal vaccines for COVID-19, influenza, RSV, pertussis, and tuberculosis. **Results:** A total of 65 studies with 229,614 participants were included in the final analysis. Mucosal COVID-19 vaccines elicited higher neutralizing antibodies compared to intramuscular vaccines (SMD = 2.48, 95% CI: 2.17–2.78 for wild-type; SMD = 1.95, 95% CI: 1.32–2.58 for Omicron), with varying efficacy by route (inhaled VE = 47%, 95% CI: 22–74%; intranasal vaccine VE = 17%, 95% CI: 0–31%). Mucosal influenza vaccines protected children well (VE = 62%, 95% CI: 30–46%, I^2^ = 17.1%), but seroconversion rates were lower than those of intramuscular vaccines. RSV and pertussis vaccines had high seroconversion rates (73% and 52%, respectively). Tuberculosis vaccines were reviewed systemically, exhibiting robust cellular immunogenicity. Safety was comparable to intramuscular vaccines or placebo, with no publication bias detected. **Conclusions:** Current evidence suggests mucosal vaccines are immunogenic, safe, and protective, particularly for respiratory diseases. This review provides insights for future research and vaccination strategies, though limitations include varying efficacy by route and study heterogeneity.

## 1. Introduction

Respiratory infectious diseases, including COVID-19, influenza, respiratory syncytial virus (RSV), pertussis, and tuberculosis, pose a significant global health threat [1]. As of 25 February 2025, COVID-19 has affected over 770 million people, resulting in 7.09 million deaths [2]. Influenza impacts around 1 billion people annually, causing 3 to 5 million severe cases and 290,000 to 650,000 deaths each year [3]. RSV, a major cause of lower respiratory infections, affects 64 million globally, leading to 160,000 deaths annually [4]. There were 14,894 confirmed pertussis cases in England in 2024 [5], while in 2023, there were 8.2 million reported new tuberculosis diagnoses, leading to and 1.25 million deaths [6].

These diseases disproportionately affect vulnerable groups, such as infants, the elderly, and those with comorbidities, emphasizing the need for effective vaccination strategies. Traditional injectable vaccination induces systemic immunity but faces challenges like needle phobia, reducing acceptance, and limited effectiveness against transmission [7]. Mucosal vaccines, delivered intranasally or via inhalation, offer non-invasive options, enhancing public willingness and inducing site-specific immunity, including secretory IgA (sIgA) and tissue-resident memory cells, potentially preventing infection at entry points [7,8].

The COVID-19 pandemic has highlighted the need for innovative approaches, with mucosal vaccines like the inhaled adenovirus type 5 (Ad5) COVID-19 vaccine and intranasal FluMist showing promise [9]. However, comprehensive evaluations across pathogens are lacking, with previous studies focusing narrowly on specific aspects, such as humoral immunogenicity, leaving gaps in assessing overall efficacy and cellular responses [10].

Given the ongoing burden of respiratory diseases and rapid advancements in mucosal vaccine research, a systematic review is timely. Recent developments, spurred by COVID-19, include new clinical trials and the exploration of adenoviral vectors, yet gaps remain, particularly for RSV, pertussis, and tuberculosis, justifying a comprehensive synthesis to inform future strategies.

While current research on mucosal vaccines mainly consists of clinical trials and systematic reviews, there is a significant absence of meta-analysis evaluation [10]. Previous systematic reviews and meta-analyses have evaluated inhaled COVID-19 vaccines, demonstrating their favorable humoral immunogenicity and safety profile. However, comprehensive analyses concentrating on intranasal COVID-19 vaccines and assessments of cellular immune responses induced by COVID-19 mucosal vaccines remain insufficient [11]. Additionally, a meta-analysis published in 2021 investigated the humoral immunogenicity and safety of influenza intranasal vaccines [12]. Nevertheless, that study had notable limitations, as it failed to evaluate mucosal and cellular immune responses, as well as vaccine efficacy. To date, no meta-analyses are available for mucosal vaccines targeting respiratory syncytial virus (RSV), pertussis, or tuberculosis. The limitation impedes a holistic assessment of how mucosal vaccines compare to traditional injectable vaccines in terms of overall efficacy. Moreover, insufficient immunogenicity and the lack of suitable delivery vehicles remain limiting factors for the clinical use of mucosal vaccines.

This systematic review and meta-analysis aim to assess the immunogenicity, safety, and protective efficacy of mucosal vaccines, providing evidence to guide future research and vaccination strategies.

## 2. Methods

### 2.1. Study Design

This systematic review adhered to Systematic Reviews and Meta-Analyses (PRISMA) [13] and Meta-analysis of Observational Studies in Epidemiology (MOOSE) guidelines (Supplementary Materials Table S1) [14]. A pre-registered protocol (PROSPERO ID: CRD420250649097) defined the research question, search strategy, inclusion/exclusion criteria, outcomes, data extraction, and synthesis.

### 2.2. Search Strategy and Selection Criteria

We searched Cochrane Central Register of Controlled Trials and PubMed up to 30 May 2025 for randomized controlled trials (RCTs), non-RCTs, and cohort studies evaluating the safety, immunogenicity, and protective efficacy of mucosal vaccines against respiratory infectious diseases. Search terms included “Vaccines”, “Inhalation”, “Intranasal”, their synonyms and MeSH terms, combined using Boolean operators (Supplementary Materials Table S2). Reference lists of included studies were screened, experts consulted, and corresponding authors contacted for missing or incomplete data.

Inclusion/exclusion criteria followed the Population, Intervention, Comparison, Outcome, and Study design (PICOS) design (Supplementary Materials Table S3) [15]. Studies were included if they (1) investigated mucosal vaccine safety, immunogenicity, or efficacy; (2) were RCTs, non-RCTs, or cohort studies; (3) focused on respiratory infectious diseases; and (4) were original English articles. Exclusion criteria included (1) non-inhaled/intranasal vaccines; (2) reviews, protocols, non-peer-reviewed, or non-full-text articles; (3) in vivo/in vitro studies; and (4) non-English articles. Two researchers independently screened titles/abstracts and full texts, resolving discrepancies with a third researcher.

### 2.3. Development of Meta-Analysis

Risk of bias was assessed as “low,” “high,” or “some concerns.” by two researchers using validated tools: the Risk of Bias-2 (RoB-2) for RCTs [16], the Newcastle–Ottawa Scale (NOS) for cohort studies [17], and the Risk of Bias in Non-Randomized Studies of Interventions (ROBINS-I) for non-RCTs [18]. RoB-2 evaluated randomization, deviations, missing data, outcome measurement, and result selection. ROBINS-I assessed confounding, selection, intervention classification, deviations, missing data, measurement, and reporting. NOS evaluated selection, comparability, and outcomes. Discrepancies were resolved through discussion with the corresponding author.

Data extraction used a pre-specified form, verified by a second researcher. Extracted data included study characteristics (author, year, country, design, period), participant characteristics (population, sample size, age, sex), intervention (comparison, dosage, vaccine composition), outcome (safety, efficacy, immunogenicity), funding, and conflicts of interest.

Immunogenicity, the primary outcome, was measured by seroconversion rates (four-fold antibody increase) and neutralizing/binding antibody geometric mean titers (nAb-GMT/bAb-GMT). Cellular immunity was assessed via T cell ELISpot IFN-γ responses and intracellular cytokine staining. Safety, the secondary outcome, was evaluated as risk ratio (RR) for adverse events. Protective efficacy, the third outcome, was calculated as vaccine efficacy (VE) using VE = (1 − RR) × 100 or 1-odds ratio (OR), with pooled VE derived from RR estimates. Dichotomous outcomes used RR with 95% CIs; continuous outcomes used standardized mean difference (SMDs) after logarithmic transformation.

### 2.4. Statistical Analysis

Meta-analysis was conducted using Stata (version 18.0) with *p* < 0.05 for significance. Pooled estimates used fixed or DerSimonian–Laird random-effects model based on heterogeneity [19], where the random-effects approach estimates between-study heterogeneity (τ^2^) using a moment-based approach with inverse-variance weighting. Heterogeneity was assessed via I^2^ (<25%: none; 25–50%: low; 50–75%: moderate; >75%: high) [20].

Subgroup analyses explored administration method, comparison group, and Omicron sub-lineages (Q-test, *p* < 0.05). Sensitivity analyses excluded individual studies to test robustness. Publication bias was evaluated using funnel plots and Egger’s regression asymmetry test, (*p* < 0.05) [21], with trim-and-fill applied if bias was detected [22].

## 3. Results

**Figure 1:**
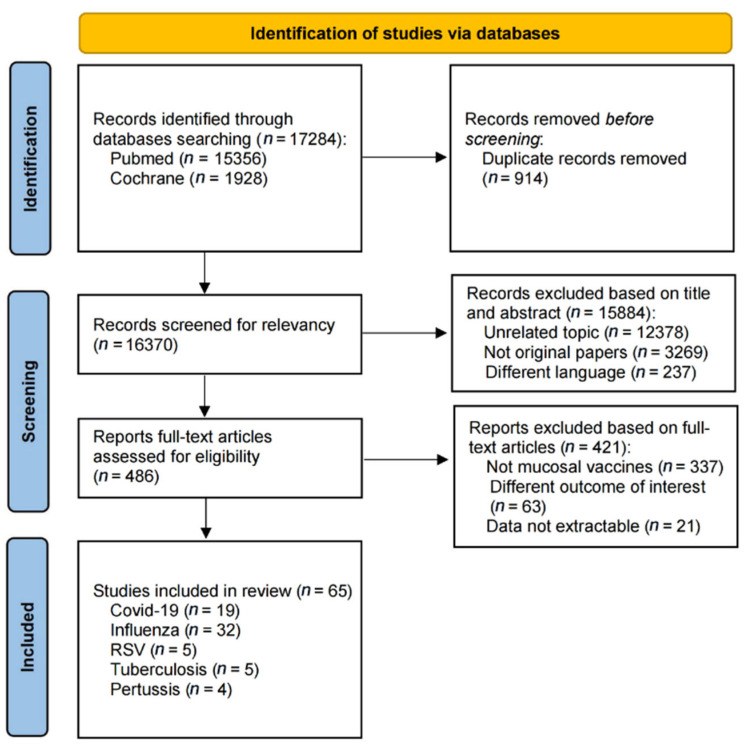
Flow chart of the selection process.

We identified 17,284 records through comprehensive database searches, including 15,356 from PubMed and 1928 records from Cochrane. After we excluded 914 duplicates, 16,370 records underwent title and abstract screening. During this phase, we excluded 12,378 records irrelevant to the topic, 3269 non-original articles, and 237 records in languages other than the target language. We then assessed 486 full-text articles for eligibility based on predefined inclusion criteria. Of these, 337 studies were excluded for not focusing on mucosal vaccines, 63 had outcomes not meeting our criteria, and 21 had data that could not be extracted. Ultimately, 65 studies were included in the systematic review, with 19 on COVID-19 [23,24,25,26,27,28,29,30,31,32,33,34,35,36,37,38,39,40,41], 32 on influenza [42,43,44,45,46,47,48,49,50,51,52,53,54,55,56,57,58,59,60,61,62,63,64,65,66,67,68,69,70,71,72,73], 5 on RSV [74,75,76,77,78], 5 on tuberculosis [79,80,81,82,83], and 4 on pertussis [84,85,86,87] (Figure 1). The same 65 studies were eligible for meta-analyses where data permitted.

## 4. Characteristics of the Included Studies

The included studies predominantly evaluated intranasal vaccines (51 studies) over inhaled vaccines (14 studies), with a focus on immunogenicity (54 studies) and safety (56 studies), while fewer assessed protective efficacy (11 studies). Randomized controlled trials were the most common design (49 studies), followed by non-randomized controlled trials (10 studies) and cohort studies (6 studies). Table 1 summarizes the main characteristics extracted, including vaccine type, target pathogen, study design, participant numbers, and outcomes measured.

## 5. The Immunogenicity of Mucosal Vaccines

**Figure 2:**
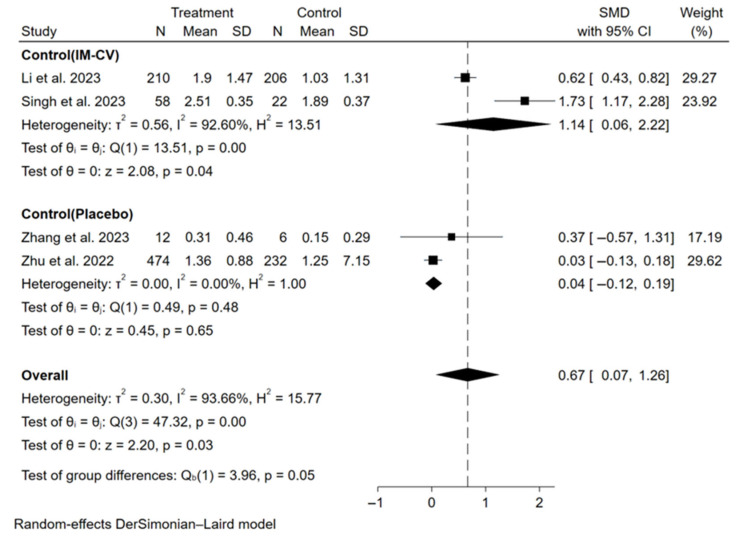
Forest plot showing the SMD of bAb-GMT of sIgA for mucosal COVID-19 vaccines. IM-CV: intramuscular COVID-19 vaccines.

**Figure 3:**
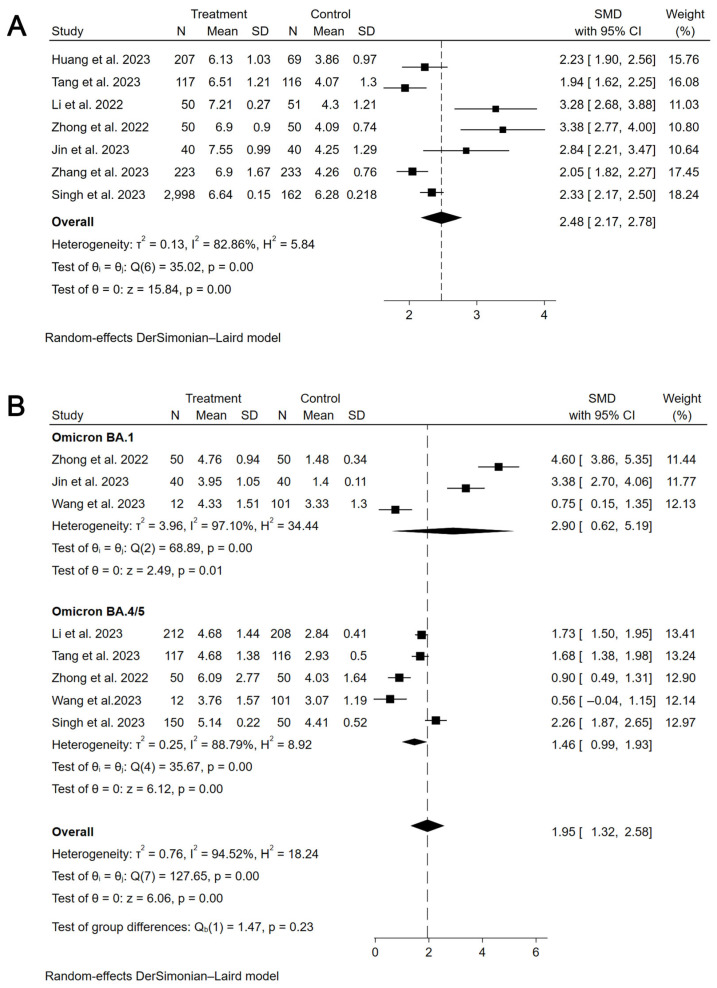
Forest plot showing the SMD of nAb-GMT for mucosal COVID-19 vaccines (wide-type and Omicron). (**A**) the SMD of nAb-GMT for mucosal COVID-19 vaccines (wide-type). (**B**) The SMD of nAb-GMT for mucosal COVID-19 vaccines (Omicron).

**Figure 4:**
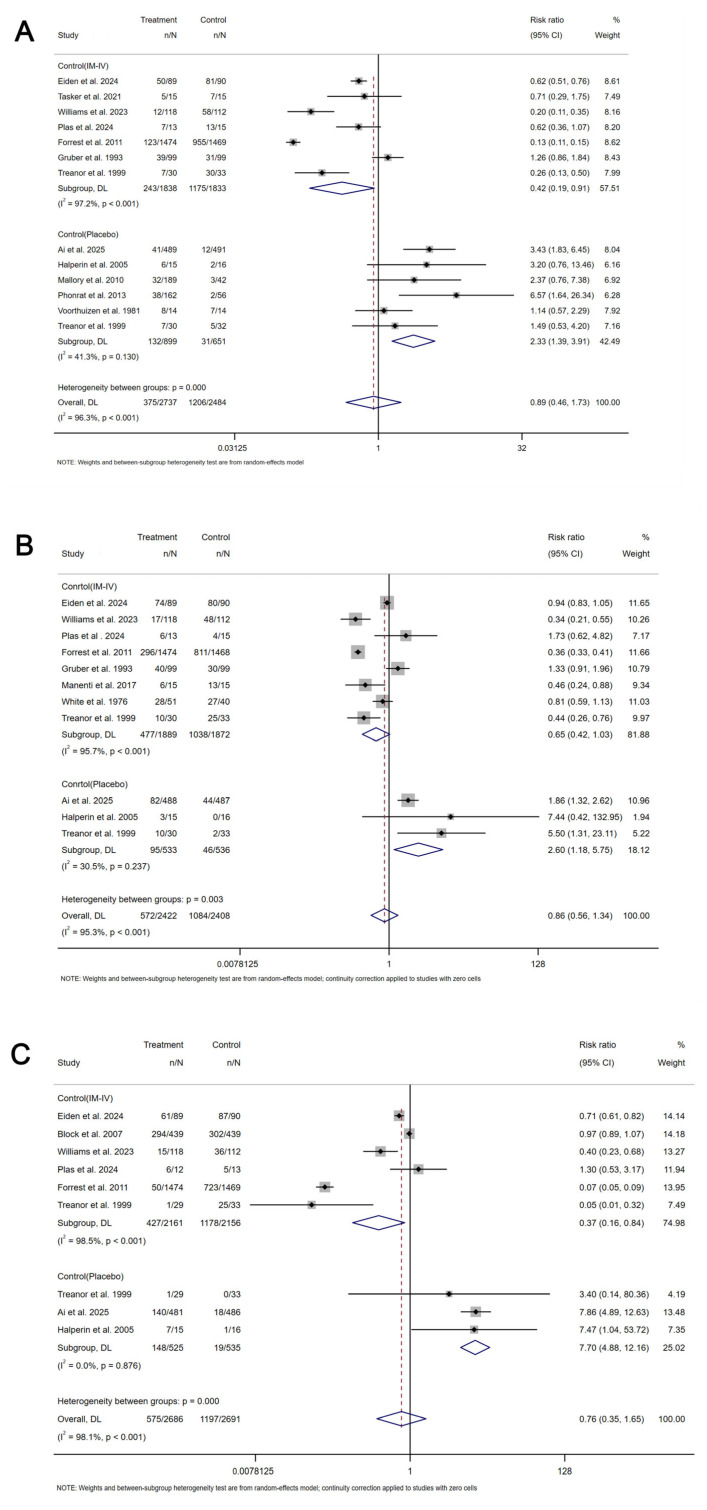
Forest plot showing the risk ratio of seroconversion rate for mucosal influenza vaccines. (**A**) The risk ratio of seroconversion rate for mucosal influenza vaccines for A/H1N1 strain. (**B**) The risk ratio of seroconversion rate for mucosal influenza vaccines for A/H3N2 strain. (**C**) The risk ratio of seroconversion rate for mucosal influenza vaccines for B strain. IM-IV: intramuscular injection inactivated influenza vaccines. n: number of incidents. N: total number of participants.

The primary assessment focused on the immunogenicity and safety of mucosal vaccines. For secretory IgA (sIgA), a meta-analysis of 10 studies (6 influenza, 4 COVID-19) showed that mucosal COVID-19 vaccines had a pooled standardized mean difference (SMD) for binding antibody geometric mean titer (bAb-GMT) of 0.67 (95% CI: 0.07–1.26, participants = 1220), with high heterogeneity (I^2^ = 93.7%) compared to controls (Figure 2). For mucosal influenza vaccines, pooled seroconversion rates were 65% (95% CI: 41–90%, participants = 229, I^2^ = 92.4%,) for A/H1N1, 45% (95% CI: 19–70%, participants = 252, I^2^ = 92.2%) for A/H3N2, and 44% (95% CI: 13–75%, participants = 151, I^2^ = 86.4%) for B strain (Supplementary Materials Figures S3–S5). For humoral immunity, a meta-analysis of 37 studies showed mucosal COVID-19 vaccines had a pooled SMD for neutralizing antibody geometric mean titer (nAb-GMT) of 2.48 (95% CI: 2.17–2.78, participants = 4406, I^2^ = 82.9%) for the wild-type strain and 1.95 (95% CI: 1.32–2.58, participants = 1359, I^2^ = 94.5%) for the Omicron strain, both higher than intramuscular inactivated vaccines (Figure 3). For mucosal influenza vaccines, the pooled relative risk (RR) of seroconversion rates for A/H1N1 was 0.89 (95% CI: 0.46–1.73, participants = 5191, I^2^ = 96.3%) compared to intramuscular vaccines, though higher than placebo (RR = 2.33, 95% CI: 1.39–3.91, participants = 1550, I^2^ = 41.3%). Similar trends were observed for A/H3N2 (RR = 0.65, 95% CI: 0.42–1.03, participants = 3761, I^2^ = 95.7%) compared to intramuscular vaccines, though higher than placebo (RR = 2.60, 95% CI: 1.18–5.75, participants = 1069, I^2^ = 30.5%), and for B strain (RR = 0.37, 95% CI: 0.16–0.84, participants = 4317, I^2^ = 98.5%) compared to intramuscular vaccines, though higher than placebo (RR = 7.70, 95% CI: 4.88–12.16, participants = 1060, I^2^ = 0.0%) (Figure 4). Pooled seroconversion rates for RSV and pertussis vaccines were 73% (95% CI: 51–94%, participants = 166, I^2^ = 88.1%, *p* < 0.0001) and 52% (95% CI: 23–82%, participants = 78, I^2^ = 85.6%), respectively (Supplementary Materials Figures S6 and S7). Cellular immunity showed mucosal vaccines for COVID-19 and influenza eliciting robust Th1 and CD8+ T cell responses, with tuberculosis vaccines (four studies) enhancing airway-resident memory T cells (Table 2).

## 6. The Safety of Mucosal Vaccines

In the secondary assessment, the safety analyses, based on 25 studies (Table 3), showed mucosal COVID-19 vaccines had a comparable risk of adverse events to intramuscular vaccines, including fever (RR = 2.24, 95% CI: 1.08–4.64, participants = 11,130, I^2^ = 0.0%), myalgia (RR = 1.12, 95% CI: 0.34–3.72, I^2^ = 0.0%), cough (RR = 2.86, 95% CI: 0.82–10.03, I^2^ = 0.0%), and sore throat (RR = 3.93, 95% CI: 1.08–14.27, I^2^ = 0.0%). For mucosal influenza vaccines, risks were higher than intramuscular vaccines for cough (RR = 2.27, 95% CI: 0.80–6.41, participants = 13,475, I^2^ = 85.7%) and nasal congestion (RR = 2.78, 95% CI: 0.79–9.77, I^2^ = 94.2%) but similar to placebo.

## 7. The Vaccine Effectiveness of Mucosal Vaccines

**Figure 5:**
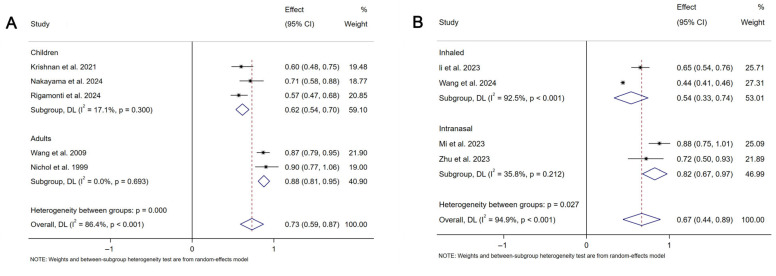
Forest plot showing the vaccine efficacy for mucosal COVID-19 vaccines and mucosal influenza vaccines. (**A**) The vaccine efficacy for mucosal COVID-19 vaccines. (**B**) The vaccine efficacy for mucosal influenza vaccines.

In the third assessment, a meta-analysis of nine studies revealed a pooled vaccine effectiveness (VE) for mucosal COVID-19 vaccines of 35% (95% CI: 4–56%, I^2^ = 96.6%,), with inhalation at 47% (95% CI: 22–74%, I^2^ = 94.5%) and intranasal spray at 17% (95% CI: 0–31%, I^2^ = 34.1%). For influenza, pooled VE was 27% (95% CI: 13–41%, I^2^ = 86.4%), with 62% (95% CI: 30–46%, I^2^ = 17.1%) in children and 12% (95% CI: 5–19%, I^2^ = 0%) in adults (Figure 5).

## 8. Risk of Bias

Among randomized controlled trials, 17 of 49 had high risk due to selection and performance bias (Supplementary Materials Tables S4–S6). One of six cohort studies scored 4 on the NOS scale (high risk), with five at low risk (score ≥ 7). Eight of ten non-randomized trials had moderate risk due to confounding and selection bias, with two at low risk (Supplementary Materials Figures S1 and S2).

## 9. Sensitivity Analyses and Publication Bias

Heterogeneity was high in most analyses (I^2^ = 0–96.6%). Sensitivity analyses showed no single study significantly altered pooled estimates (Supplementary Materials Figures S8–S25). Egger’s test indicated no publication bias (Supplementary Materials Table S7).

## 10. Discussion

This meta-analysis and systematic review, the first to comprehensively assess mucosal vaccines against respiratory infectious diseases, analyzed 65 studies—19 on COVID-19, 32 on influenza, 5 on RSV, 5 on tuberculosis, and 4 on pertussis—offering critical insights into their immunogenicity, safety, and protective efficacy. Our findings reveal that mucosal vaccines induce robust mucosal immunity, particularly through secretory IgA, demonstrate a favorable safety profile with no increased risk of adverse events compared to intramuscular vaccines or placebo, and provide valid protective efficacy, with vaccine effectiveness (VE) of 35% (95% CI: 4–56%) for COVID-19 and 27% (95% CI: 13–41%) for influenza.

Our hypothesis posited that mucosal vaccines would elicit strong mucosal immunity and offer comparable or superior protection to intramuscular vaccines. The results support this: mucosal COVID-19 vaccines significantly increased sIgA levels (SMD = 0.67, 95% CI: 0.07–1.26) and neutralizing antibody titers against wild-type (SMD = 2.48, 95% CI: 2.17–2.78) and Omicron strains (SMD = 1.95, 95% CI: 1.32–2.58) compared to intramuscular vaccines. Mucosal influenza vaccines achieved high sIgA seroconversion rates (e.g., 65% for A/H1N1, 95% CI: 41–90%), though their neutralizing antibody levels were lower than intramuscular vaccines (RR = 0.42, 95% CI: 0.19–0.91) but exceeded placebo (RR = 2.33, 95% CI: 1.39–3.91). Limited data on RSV and pertussis showed moderate seroconversion (73% and 52%, respectively), while tuberculosis vaccines enhanced airway-resident T cells. These findings align with prior research, such as FluMist’s effectiveness in children [88], which our subgroup analysis confirmed with higher VE in pediatric populations (62% vs. 12% in adults).

The study’s strengths include its broad scope across multiple pathogens and outcomes, the novel use of sIgA as a mucosal immunity marker, and the inclusion of diverse study designs (49 RCTs, 10 non-RCTs, 6 observational), enhancing generalizability. However, limitations temper our conclusions. High heterogeneity (I^2^ up to 96.6%) arose from varied participant demographics, vaccine platforms, and administration routes, though sensitivity analyses confirmed robustness. Bias is a concern, with eight studies at high risk and ten with moderate risk, necessitating cautious interpretation. Publication bias was not evident, but sparse data on cellular immunity, long-term efficacy, and special populations (e.g., immunocompromised, pregnant) limit the findings’ scope. Most participants were healthy, reducing applicability to vulnerable groups.

No major controversies emerged, but discrepancies with other reviews exist. Some report lower systemic antibody responses for mucosal vaccines [89], consistent with our influenza findings, possibly due to differing study designs or analytical methods—our inclusion of observational data contrasts with RCT-focused reviews.

However, mucosal vaccination offers a strategic advantage for preventing infections but struggles with unique biological and technological constraints. Conventional live-attenuated vaccines pose safety risks, whereas modern alternatives often fail to elicit sufficient mucosal immune responses without tailored adjuvants or delivery vehicles. Overcoming these challenges requires innovative approaches to harmonize antigen presentation, adjuvant activity, and tissue-specific targeting at mucosal sites.

Future research should prioritize large-scale, high-quality trials to assess long-term efficacy, cellular immunity, and safety across diverse populations. Optimizing administration routes (e.g., inhaled vs. intranasal) and vaccine platforms also warrants investigation, given inhaled COVID-19 vaccines’ superior VE.

## 11. Conclusions

Collectively, the evidence suggests mucosal vaccines are a promising tool for respiratory disease prevention, leveraging mucosal immunity at pathogen entry points. Their non-invasive delivery could enhance uptake, particularly in children, and reduce healthcare burdens. Clinically, their significance lies in potentially controlling pandemics, though data gaps for RSV, tuberculosis, and cellular immunity require attention. For policy and practice, these findings advocate continued investment in mucosal vaccine development to bolster global health security, especially for pediatric and high-risk groups.

## Figures and Tables

**Table 1:** Trials of vaccine immunogenicity, safety, and efficacy meeting inclusion criteria.

Study	Country	Study Design	No. of Research Centers	Infectious Disease	Administration	Vaccines	Comparison	Population Characteristics	Sample Size	Safety	Immunogenicity	Protective Efficacy
Li et al. (2023) [23]	China	A multicenter, open-label phase 3 trial.	Multicenter	COVID-19	Inhalation	Ad5-nCoV	Inactivated vaccine	Men: 5738 Women: 4529 Median age: 53 years (18–92)	Safety cohort Aerosolized Ad5-nCoV: 10,059 Immunology sub-cohort Aerosolized Ad5-nCoV: 212 Inactivated vaccine: 208	√	√	√
Li et al. (2022) [24]	China	A randomized, open label, single-center, controlled trial.	Single-center	COVID-19	Inhalation	Ad5nCoV	CoronaVac	Chinese adult: ≥18 y	Low-dose group: 140 High-dose group: 140 CoronaVac group: 140	√	√	N.A.
Wang et al. (2023) [25]	China	A prospective cohort, open-label study.	Multicenter	COVID-19	Inhalation	BBIBP-CorV+ Convidecia or BBIBP-CorV+ aerosolized Convidecia	CoronaVac+ aerosolized Convidecia	Age: 19–59 y Mean age: 37.5 y (SD 10.3 years)	BBIBP-CorV+ Convidecia: 101 BBIBP-CorV+ aerosolized Convidecia: 12 CoronaVac + aerosolized Convidecia: 23	N.A.	√	N.A.
Sun et al. (2024) [34]	China	An open-label, single-center, investigator-initiated trial.	Single-center	COVID-19	Intranasal spray	NB2155	N.A.	Age: 18–59 y	I: 128	√	√	N.A.
Wu et al. (2021) [26]	China	A single-center, open-label, randomized phase 1 trial.	Single-center	COVID-19	Inhalation	Ad5-nCoV	N.A.	Age: ≥18 y	HDmu group: 26 LDmu group: 26 MIX group: 26 1Dim group: 26 2Dim group: 26	√	√	N.A.
Dodaran et al. (2023) [35]	Iran	A single-center, randomized, double-blind controlled, dose-finding trial.	Single-center	COVID-19	Intranasal spray	Spike Protein COVID-19 vaccine (RCP)	Placebo	Healthy men and non-pregnant women age: 18–55 y	Placebo: 30 Vac.5: 30 Vac.10: 30 Vac.20: 30	√	√	N.A.
Zhang et al. (2023) [27]	China	A non-randomized, open-label and parallel controlled phase 4 trial.	Multicenter	COVID-19	Inhalation	Ad5-nCoV or ZF2001, CoronaVac	N.A.	Healthy people: 19–25 y	Ad5-nCoV-IM: 229 Ad5-nCoV-IH: 223 ZF2001: 219 CoronaVac: 233	√	√	N.A.
Madhavan et al. (2022) [36]	UK	An open-label phase 1 clinical trial.	Single-center	COVID-19	Intranasal spray	IN ChAdOx1 nCoV-19	N.A.	Healthy people: 18–53 y	Group 1 5 × 10^9^vp ChAdOx1nCOV-19IN: 6 Group 2 5 × 10^10^vp ChAdOx1nCOV-19IN: 12 Group 3 2 × 10^10^vp ChAdOx1nCOV-19IN: 12 Group 4 Previous ChAdOx1nCoV-19IM: 6 Group 5 Previous BNT162b2IM: 6	√	√	N.A.
Mi et al. (2024) [37]	China	A prospective cohort study.	Single-center	COVID-19	Intranasal spray	dNS1-RBD	N.A.	Healthy people: ≥18 y	I: 536 C: 811	N.A.	N.A.	√
Huang et al. (2023) [28]	China	A randomized, open-label, parallel-controlled, non-inferiority study.	Multicenter	COVID-19	Inhalation	Ad5-nCoV or BBIBP-CorV or CoronaVac	N.A.	Children: 6–12 y Adolescents: 13–17 y	Children AAd5: 110 IMAd5: 35 Inactivated vaccine: 35 Adolescents AAd5: 110 IMAd5: 35 Inactivated vaccine: 35	√	√	N.A.
Tang et al. (2023) [29]	China	An open-label, parallel-controlled, phase 4, randomized trial.	Multicenter	COVID-19	Inhalation	Ad5-nCoV or CoronaVac	N.A.	Healthy people: ≥18 y	Aerosolized Ad5-nCoV: 117 Intramuscular Ad5-nCoV: 120 CoronaVac: 119	√	√	N.A.
Singh et al. (2023) [38]	India	A randomized, open-label, multicenter trial.	Multicenter	COVID-19	Intranasal spray	iNCOVACC or Covaxin	N.A.	Healthy men and non-pregnant women age: ≥18 y	BBV154: 2989 Covaxin: 161	√	√	N.A.
Zhong et al. (2022) [30]	China	A randomized, open-label, single-center trial.	Single-center	COVID-19	Intranasal spray	Ad5-nCoV or CoronaVac	N.A.	Group A median age: 41.62 y (41.62 ± 9.10). Group B median age: 40.90 y (40.90 ± 9.77). Group C median age: 42.12 y (42.12 ±8.56).	Group A low I-I-Ad5: 50 Group B high I-I-Ad5: 50 Group C I-I-I: 50	N.A.	√	N.A.
Jin et al. (2023) [31]	China	A randomized, open-label, single-center trial.	Single-center	COVID-19	Inhalation	Ad5-nCoV or CoronaVac	N.A.	Healthy people: ≥18 y	Low-dose group: 140 High-dose group: 139 CoronaVac group: 140	√	√	N.A.
Wang et al. (2023) [25]	China	A prospective cohort study.	Multicenter	COVID-19	Inhalation	Convidecia Air	N.A.	Group A: 18–59 y Group B: ≥60 y Average age of infected individuals: 46.1 ± 15.5 y Average age of uninfected individuals: 46.3 ± 15.5 y	FAS-7d N: 13,600 FAS-14d N: 6576	√	N.A.	√
Xu et al. (2023) [33]	China	A randomized, double blinded, parallel controlled trial.	Single-center	COVID-19	Inhalation	Ad5-nCoVO-IH or Ad5-nCoV/O-IH or Ad5-nCoV-IH	N.A.	Healthy people: ≥18 y	Ad5-nCoVO IH group: 150 Ad5-nCoV/O-IH group: 151 Ad5-nCoV IH group: 150	√	√	N.A.
Zhang et al. (2023) [39]	China	A phase 1, randomized, double-blinded, placebo-controlled, dose escalation study.	Single-center	COVID-19	Intranasal spray	DelNS1-nCoV-RBD LAIV	Placebo	Healthy people: 18–55 y	High-dose: 12 Low-dose: 11 Placebo: 6	√	√	N.A.
Zhu et al. (2022) [40]	China	A single-center, double-blind, randomized, placebo-controlled study.	Single-center	COVID-19	Intranasal spray	dNS1-RBD	Placebo	Healthy people: ≥18 y	Phase 1 trial I: 51 C: 12 Phase 2 trial I: 485 C: 239 Phase 2 extension trial (subgroup) I: 148 C: 149	√	√	N.A.
Zhu et al. (2023) [41]	China	A multicenter, randomized, double-blind, placebo-controlled, case-driven, and adaptive design phase 3 trial.	Multicenter	COVID-19	Intranasal spray	dNS1-RBD	Placebo	Healthy men and non-pregnant women age: ≥18 y	Participants without a COVID-19 vaccination history I: 6910 C: 6904 Participants with a COVID-19 vaccination history I: 8586 C: 8590	√	N.A.	√
Ambrose et al. (2013) [42]	USA	A randomized, placebo-controlled study.	Multicenter	Influenza	Intranasal spray	LAIV	Placebo	Healthy people: 18–64 y	SA-LAIV: 2026 HCP-LAIV: 805 Placebo: 1420	√	√	N.A.
Pan et al. (2020) [43]	China	A multicenter, randomized controlled, double-blind phase 2 trial.	Multicenter	Influenza	Intranasal spray	LTh(αK)	Trivalent inactivated influenza virus antigens without adjuvant.	Healthy people: 20–70 y	Group 1 22.5 μg HA + 30 μg LTh(αK) adjuvant doses: 141 Group 2 22.5 μg HA + 45 μg LTh(αK) adjuvant doses: 139 Group 3 22.5 μg HA: 72	√	√	N.A.
Halperin et al. (2005) [44]	Canada	A randomized, placebo-controlled, dose-escalating clinical phase 1 trial.	Multicenter	Influenza	Intranasal spray	Inactivated trivalent influenza virus vaccine	Placebo	Healthy people: 18–50 y	N: 61	√	√	N.A.
Kiseleva et al. (2020) [45]	Russia	A randomized, double-blind, placebo-controlled study.	Multicenter	Influenza	Intranasal spray	H7N9 LAIV	Placebo	Healthy men and non-pregnant women age: 18–49 y	I: 30 C: 8	√	√	N.A.
Forrest et al. (2011) [46]	South Africa	A prospective, randomized, open-label, multicenter trial.	Multicenter	Influenza	Intranasal spray	LAIV or TIV	N.A.	Healthy people: ≥60 y	LAIV: 1508 TIV: 1501	√	√	N.A.
Manenti et al. (2017) [47]	Norway	A randomized, double-blind study.	Single-center	Influenza	Intranasal spray	IIV3 or LAIV3	N.A.	Group A: <5 y Group B: 10–17 y Group C: ≥18 y Group D: ≥18 y	Group A Children <5: 15 Group B Children 10–17 y: 14 Group C LAIV3 vaccinated adults: 15 Group D IIV3 vaccinated adults: 15	N.A.	√	N.A.
Nichol et al. (1999) [48]	USA	A randomized, double-blind, placebo-controlled trial.	Multicenter	Influenza	Intranasal spray	LAIV	Placebo	Healthy people: 18–64 y	I: 3041 C: 1520	N.A.	√	√
Mallory et al. (2010) [49]	USA	Two randomized, double-blind, placebo-controlled studies.	Multicenter	Influenza	Intranasal spray	H1N1 LAIV	Placebo	Children group: 2–17 y Adults group: 18–49 y	Children (2–17 y) I: 261 C: 65 Adults (18–49 y) I: 240 C: 60	√	√	N.A.
Block et al. (2007) [50]	USA	A prospective, phase 3, randomized, double-blind, multicenter trial.	Multicenter	Influenza	Intranasal spray	CAIV-T or frozen LAIV	N.A.	Healthy people: 5–49 y	Two-dose group (ages 5 to 8 y) CAIV-T: 186 LAIV: 190 One-dose group (ages 9 to 49 y) CAIV-T: 285 LAIV: 281	√	√	N.A.
Gruber et al. (1993) [51]	USA	A randomized, double-blind study.	Single-center	Influenza	Nose drop or Inhalation	Bivalent cold-adapted(ca) influenza A vaccine	N.A.	Healthy people: ≥18 y	ND: 97 LPA: 98	√	√	N.A.
Hammitt et al. (2009) [52]	USA	An open-label, 2-arm study.	Multicenter	Influenza	Intranasal spray	TIV or CAIV	N.A.	Healthy people: 18–45 y	CAIV: 10 TIV: 5	N.A.	√	N.A.
Tong et al. (2024) [53]	USA	A randomized, double-blind study.	Multicenter	Influenza	Intranasal spray	IIV/Fluzone	LAIV/FluMist	LAIV/FluMist Median (range) age: 26 (18–49). IIV/Fluzone Median (range) age: 28 (20–51)	IIV/Fluzone: 63 LAIV/FluMist: 94	N.A.	√	N.A.
Krishnan et al. (2021) [54]	India	A 2-year, triple (participant–observer–analyst)-blind, community-based vaccine trial.	Multicenter	Influenza	Intranasal spray	LAIV or IIV or IPV	Placebo	Healthy children: 2–10 y	LAIV: 1015 IIV: 1010 placebo: 507 IPV: 509	√	N.A.	√
Ai et al. (2025) [55]	China	A randomized, double-blind, placebo-controlled trial.	Multicenter	Influenza	Intranasal spray	LAIV	Placebo	Healthy children and adolescents: 3–17 y	I: 1500 C: 1500	√	√	N.A.
Phonrat et al. (2013) [56]	Thais	A randomized, double blind, placebo-controlled study.	Multicenter	Influenza	Intranasal spray	H1N1 LAIV candidate strain	Placebo	Healthy people: 12–75 y	I: 271 C: 92	√	√	N.A.
Williams et al. (2023) [57]	USA	A randomized controlled clinical trial.	Multicenter	Influenza	Intranasal spray	ccIIV4	LAIV4	Healthy children and adolescents: 4–21 y	ccIIV4: 112 LAIV4: 118	√	√	N.A.
Nakayama et al. (2024) [58]	Japan	A randomized, double-blind, phase 3 study.	Multicenter	Influenza	Intranasal spray	MEDI3250	Placebo	Healthy children and adolescents: 2–18 y	I: 608 C: 302	√	N.A.	√
Pitisuttithum et al. (2017) [59]	Thais	A randomized, double-blind, placebo-controlled study.	Single-center	Influenza	Intranasal spray	LAIV H5N2 and H5N1 booster vaccine	Placebo	Healthy people: 18–49 y	Part 1 I: 101 C: 51 Part 2 I: 40 C: 20	√	√	N.A.
Rudenko et al. (2014) [60]	Russia	A randomized, double-blind, and placebo-controlled phase 1 study.	Single-center	Influenza	Intranasal spray	H7N3 LAIV	Placebo	Healthy people: 18–49 y	I: 30 C: 10	√	√	N.A.
Rudenko et al. (2015) [61]	Russia	A randomized, double-blind, placebo-controlled study.	Single-center	Influenza	Intranasal spray	A/H5N2 LAIV	Placebo	Healthy people: 18–49 y	I: 30 C: 10	√	√	N.A.
Rudenko et al. (2016) [62]	Russia	A phase 1, double-blind, randomized, placebo-controlled trial.	Single-center	Influenza	Intranasal spray	H7N9 live attenuated influenza vaccine (LAIV)	Placebo	Healthy adult men and non-pregnant women: 18–49 y.	I: 30 C: 10	√	√	N.A.
Eiden et al. (2024) [63]	USA	A randomized, double-blind, double-dummy phase 1b trial.	Multicenter	Influenza	Intranasal spray	H3N2M2SR vaccine plus placebo or H3N2 M2SR vaccine plus Fluzone HD or Fluzone HD plus placebo	Placebo	Healthy people: 65–85 y	H3N2M2SR plus placebo: 89 H3N2 M2SR plus Fluzone HD: 94 Fluzone HD plus placebo: 92 Placebo: 30	√	√	N.A.
Speroni et al. (2005) [64]	USA	An overarching survey.	Single-center	Influenza	Intranasal spray	Fluzone or FluMist	Unvaccinated	The population drawn from hospital employees, volunteers, physicians and students	Fluzone: 201 FluMist: 63 unvaccinated: 77	√	N.A.	N.A.
Tasker et al. (2021) [65]	USA	A phase 2 randomized, double-blind, placebo-controlled, single ascending-dose study.	Single-center	Influenza	Intranasal spray	NasoVAX	Placebo	Healthy people: 18–49 y	I: 45 C: 15	√	√	N.A.
Treanor et al. (1999) [66]	USA	A randomized double-blind, placebo-controlled trial.	Multicenter	influenza	Intranasal spray	CAIV-T or TIV	Placebo	Healthy people: 18–45 y	CAIV-T: 36 TIV: 33 Placebo: 34	√	√	√
Li et al. (2022) [67]	China	A randomized, double-blind, placebo-controlled study.	Single-center	Influenza	Intranasal spray	LAIV	Placebo	Group A: Healthy people ≥18 y Group B: Healthy people: 3–17 y	Group A I: 30 C: 10 Group B I: 30 C: 10	√	N.A.	N.A.
Voorthuizen et al. (1981) [68]	The Netherlands	A double-blind placebo-controlled study.	N.A.	Influenza	Intranasal spray	Live influenza A vaccine	Placebo	Healthy people: 19–28 y (mean age 22.5 y)	I: 14 C: 14	√	√	N.A.
Vesikari et al. (2015) [69]	Finland	A randomized, double-blind, placebo-controlled study.	Multicenter	Influenza	Intranasal spray	CAIV-T	Placebo	Healthy infants: 6–24 w Gestational age: ≥37 w Birth weight: ≥2500 g	6-to < 16-wk Cohort group I: 31 C: 28 16-to < 24-wk Cohort group I: 30 C: 31	√	N.A.	N.A.
Plas et al. (2024) [70]	The Netherlands	A first-in-human, randomized, double-blind, controlled, dose-escalation study.	Single-center	Influenza	Intranasal spray	FluGEM	Unadjuvanted TIV only	Part 1: Healthy people: 18–49 y Part 2: Healthy people: ≥65 y	Age 18–49 Control group: 15 1.25 mgFluGEM: 15 2.5 mgFluGEM: 15 5.0 mgFluGEM: 15 Age ≥ 65 y Control group: 15 1.25 mg FluGEM: 15	√	√	N.A.
Wang et al. (2009) [71]	USA	A statistical analysis of the national defense medical testing system.	Single-center	Influenza	N.A.	LAIV or TIV	Unvaccinated	Healthy people: 17–49 y (Army: 16–43 y; Air Force: 16–28 y; Marine Corps: 16–30 y; Navy: 16–36 y).	N.A.	N.A.	N.A.	√
Rigamonti et al. (2024) [72]	Italy	A retrospective observational cohort study.	Single-center	Influenza	N.A.	LAIV-4 or IIV	N.A.	Healthy children: 2–14 y enrolled in the Pedianet database.	N.A.	N.A.	N.A.	√
Gasparini et al. (2021) [73]	Italy	An observational study.	Multicenter	Influenza	Intranasal spray	Fluenz Tetr	N.A.	Healthy children and adolescents: 2–17 y Preschoolers: 2–5 y School-age: 6–10 y Adolescents: 11–17 y	Preschoolers: 1924 School-age: 1179 Adolescents: 116	√	N.A.	N.A.
Green et al. (2019) [74]	UK	An open-label, dose escalation, phase 1 clinical trial.	Multicenter	RSV	Intranasal spray	MVA-RSV or PanAd3-RSV	Unvaccinated	Healthy people: 18–50 y	I: 24 C: 6	√	√	N.A.
Verdijk et al. (2020) [75]	The Netherlands	A double-blinded, randomized, placebo-controlled, parallel-group, single-dose study.	Single-center	RSV	Intranasal spray	RSVΔG	Placebo	Healthy people: 18–50 y	I: 36 C: 12	√	√	N.A.
Cunningham et al. (2022) [76]	USA	A randomized, double-blind, placebo-controlled study.	Multicenter	RSV	Intranasal spray	RSV/ΔNS2/Δ1313/I1314L or RSV/276	Placebo	Median age: 12.5 y Interquartile range: 8.0–15.0 y	RSV/ΔNS2/Δ1313/I1314L: 25 RSV/276: 25 Placebo: 12	√	√	N.A.
Karron et al. (2023) [77]	USA	A randomized, double-blind, placebo-controlled study.	Single-center	RSV	Nose drops	RSV/6120/ΔNS2/1030s	Placebo	RSV-seropositive children: 12–59 m RSV-seronegative children: 6–24 m	RSV-seropositive children I: 10 C: 5 RSV-seronegative infants I: 20 C: 10	√	√	N.A.
Spearman et al. (2023) [78]	USA	A phase 1 clinical trial.	Multicenter	RSV	Intranasal spray	PIV5-RSV	N.A.	Group 1: Healthy people: 33–59 y Group 2: Healthy people: 61–75 y	Group 1 Planned participant ages 18 to 59 years: 15 Group 2 Planned participant ages 60 to 75 years: 15	√	√	N.A.
Creech et al. (2022) [84]	USA	A phase 2a, single-center, randomized, partially blind, placebo-controlled clinical trial.	Multicenter	Pertussis	Intranasal spray	BPZE1	Placebo	Healthy adult men and non-pregnant women: 18–49 y.	10^7^ CFU BPZE1 via VaxINator device: 15 10^9^ CFU BPZE1 via VaxINator device: 15 Placebo via VaxINator device: 15 10^9^ CFU BPZE1 via a needleless tuberculin syringe: 5	√	√	N.A.
Thorstensson et al. (2014) [85]	Sweden	A double-blind, placebo-controlled, dose-escalating study.	Single center	Pertussis	Intranasal spray	BPZE1	N.A.	Healthy people: 19–31 y	Group 1 I: 12 C: 4 Group 2 I: 12 C: 4 Group 3 I: 12 C: 4	√	√	N.A.
Jahnmatz et al. (2020) [86]	Sweden	A phase 1b, double-blind, randomized, placebo-controlled, dose-escalation study.	Single center	Pertussis	Intranasal spray	BPZE1	N.A.	Healthy people: 18–32 y	Group 1 I: 12 C: 4 Group 2 I: 12 C: 4 Group 3 I: 12 C: 4	√	√	N.A.
Keech et al. (2023) [87]	USA	A randomized, double-blind phase 2b study.	Multicenter	Pertussis	Intranasal spray	BPZE1 or Tdap	N.A.	Healthy people: 18–50 y	BPZE1–BPZE1: 92 BPZE1–placebo: 92 Tdap–BPZE1: 46 Tdap–placebo: 50	√	√	√
Audran et al. (2024) [79]	Swiss	A randomized, double-blind, controlled phase 1 study.	Single-center	Tuberculosis	Inhalation	ChAdOx1-85A and saline placebo	ChAdOx1-85A	Healthy people: 18–55 y	1 × 10^9^ Group: 3 5 × 10^9^ Group: 3 1 × 10^10^ Group: 3 Aerosol group: 10 Intramuscular group: 10 Naïve aerosol group: 10	√	√	N.A.
Thomas et al. (2019) [80]	UK	A phase 1 randomized, blinded clinical trial.	Multicenter	Tuberculosis	Inhalation	MVA85A	N.A.	Healthy people: 21–42 y	Group 1 aerosol (D0)-intradermal (D28): 12 Group 2 intradermal (D0)-aerosol (D28): 12 Group 3 intradermal (DO)-intradermal (D28): 12	√	√	N.A.
Jeyanathan et al. (2022) [81]	Canada	An open-label phase I trial.	Single-center	Tuberculosis	Inhalation	AdHu5Ag85A	N.A.	Healthy people: 18–55 y	LD group: 11 HD group: 11 IM group: 9	√	√	N.A.
Satti et al. (2024) [82]	UK	A controlled human infection trial consisting of a dose escalation trial followed by a single-blind, randomized, controlled, phase 1 trial.	Multicenter	Tuberculosis	Inhalation	BCG	N.A.	Healthy people: 18–50 y	Group 1 Aerosol BCG, dose escalation: 6 Aerosol BCG: 4 Intradermal BCG: 3 Group 2 Aerosol BCG, dose escalation: 9 Aerosol BCG: 12 Intradermal BCG: 12	√	√	N.A.
Satti et al. (2014) [83]	UK	A phase 1, double-blind, randomized controlled trial.	Multicenter	Tuberculosis	Inhalation	MVA85A	N.A.	Healthy people: 18–50 y	Group A aerosol MVA85A: 12 Group B intradermal MVA85A: 12	√	√	N.A.

I: intervention; HDmu: high-dose mucosal; LDmu: low-dose mucosal; MIX: mixed vaccination; 1Dim: 1 dose intramuscular; 2Dim: 2 dose intramuscular; Vac.5: vaccine at 5 μg/200 μL; Vac.10: vaccine at 10 μg/200 μL; Vac.20: vaccine at 20 μg/200 μL; IM: intramuscular; IH: inhalation; C: comparison; A: aerosolized; I-I-Ad5: inactivated-inactivated-Ad5; I-I-I: inactivated-inactivated-inactivated; FAS: full analysis set; N: total umber; SA: self-administration; HCP: administration by health care professionals; ND: nose drop; LPA: nasal delivery by large particle aerosol; N.A.: not applicable.

**Table 2:** The summary of the immunogenicity of tuberculosis mucosal vaccines.

Study	Study Design	Mucosal Vaccines	Compared Groups	Immunogenicity Results
Satti et al. (2014) [83]	Phase I, double-blind, RCT	MVA85A (modified vaccinia ankara vector tuberculosis vaccine)	Intradermal vaccination	MVA85A elicited Ag85A-specific CD4 T cells in bronchoalveolar (BAL) lavage fluid in both the inhaled and intradermal vaccination groups, with a more significant response noted in the inhaled group.
Thomas et al. (2019) [80]	Phase I, RCT	MVA85A (modified vaccinia ankara vector tuberculosis vaccine)	Intradermal vaccination	MVA85A delivered through intradermal injection solely induced the synthesis of serum Ag85A antibodies, while inhaled MVA85A provoked considerably elevated levels of Ag85A-specific CD4+ and CD8+ T-cell cytokines in the pulmonary mucosa compared to intradermal immunization.
Audran et al. (2024) [79]	Phase I, RCT, double-blind	ChAdOx1-85A (chimpanzee adenovirus vector tuberculosis vaccines)	Intramuscular vaccination	Inhaled ChAdOx1-85A induced robust lung mucosal and systemic Ag85A-specific T-cell responses compared to intramuscular ChAdOx1-85A vaccination.
Jeyanathan et al. (2022) [81]	Phase I, open-labeled	AdHu5Ag85A (human adenovirus Type 5 vector tuberculosis vaccine)	Intramuscular vaccination	Inhaled AdHu5Ag85A remarkably induced airway tissue–resident memory CD4+ and CD8+ T cells of polyfunctionality and Ag85A-specific T cell responses in blood.

**Table 3:** Safety profile of mucosal influenza vaccines compared to intramuscular vaccines and placebo.

Experimental Groups	Control Groups	Symptom	Number of Studies Included	Risk Ratio (95% CI)	Weight%	I^2^	*p*-Value
Mucosal COVID-19 vaccines	Intramuscular COVID-19 vaccines	Fever	4	2.24 (1.08, 4.64)	100	0.00%	0.929
Mucosal COVID-19 vaccines	Intramuscular COVID-19 vaccines	Myalgia	4	1.12 (0.34, 3.72)	100	0.00%	0.582
Mucosal COVID-19 vaccines	Intramuscular COVID-19 vaccines	Cough	3	2.86 (0.82, 10.03)	100	1.00%	0.364
Mucosal COVID-19 vaccines	Intramuscular COVID-19 vaccines	Sore throat	3	3.93 (1.08, 14.27)	100	0.00%	0.531
Mucosal COVID-19 vaccines	Intramuscular COVID-19 vaccines	Headache	4	2.35 (1.13, 4.87)	94.95	0.00%	0.860
Mucosal COVID-19 vaccines	Placebo	Headache	1	0.30 (0.01, 7.09)	5.05	0.00%	<0.001
Mucosal COVID-19 vaccines	Overall	Headache	5	2.12 (1.04, 4.31)	100	0.00%	0.681
Mucosal COVID-19 vaccines	Intramuscular COVID-19 vaccines	Rhinorrhea	3	4.56 (1.00, 20.68)	100	0.00%	0.875
Mucosal influenza vaccines	Placebo	Fever	8	1.00 (0.89, 1.12)	61.84	13.50%	0.325
Mucosal influenza vaccines	Intramuscular influenza vaccines	Fever	4	0.87 (0.79, 0.95)	38.16	0.00%	0.741
Mucosal influenza vaccines	Overall	Fever	12	0.95 (0.86, 1.04)	100	19.80%	0.249
Mucosal influenza vaccines	Placebo	Myalgia	4	2.32 (1.24, 4.34)	23.92	0.00%	0.969
Mucosal influenza vaccines	Intramuscular influenza vaccines	Myalgia	2	1.02 (0.87, 1.21)	76.08	0.00%	0.663
Mucosal influenza vaccines	Overall	Myalgia	6	1.28 (0.91, 1.79)	100	24.40%	0.251
Mucosal influenza vaccines	Placebo	Cough	6	0.74 (0.61, 0.89)	54.96	0.00%	0.7
Mucosal influenza vaccines	Intramuscular influenza vaccines	Cough	2	2.27 (0.80, 6.41)	45.04	85.70%	0.008
Mucosal influenza vaccines	Overall	Cough	8	1.06 (0.63, 1.78)	100	82.10%	<0.001
Mucosal influenza vaccines	Placebo	Sore throat	5	1.01 (0.84, 1.20)	60.71	0.00%	0.999
Mucosal influenza vaccines	Intramuscular influenza vaccines	Sore throat	2	1.75 (1.06, 2.87)	39.29	47.50%	0.167
Mucosal influenza vaccines	Overall	Sore throat	7	1.24 (0.96, 1.59)	100	52.10%	0.051
Mucosal influenza vaccines	Placebo	Headache	6	1.05 (0.81, 1.38)	49.86	36.30%	0.164
Mucosal influenza vaccines	Intramuscular influenza vaccines	Headache	3	1.09 (0.77, 1.55)	50.14	83.50%	0.002
Mucosal influenza vaccines	Overall	Headache	9	1.06 (0.87, 1.29)	100	60.00%	0.010
Mucosal influenza vaccines	Placebo	Nasal congestion	6	1.20 (1.02, 1.42)	51.16	0.00%	0.849
Mucosal influenza vaccines	Intramuscular influenza vaccines	Nasal congestion	2	2.78 (0.79, 9.77)	48.84	94.20%	<0.001
Mucosal influenza vaccines	Overall	Nasal congestion	8	1.66 (1.17, 2.34)	100	73.70%	<0.001
Mucosal influenza vaccines	Placebo	Rhinorrhea	6	1.11 (0.98, 1.25)	80.7	0.00%	0.535
Mucosal influenza vaccines	Intramuscular influenza vaccines	Rhinorrhea	1	4.79 (2.44, 9.38)	19.3	0.00%	<0.001
Mucosal influenza vaccines	Overall	Rhinorrhea	7	1.26 (0.68, 2.32)	100	72.30%	0.001
Mucosal RSV vaccines	Placebo	Fever	5	1.40 (0.56, 3.47)	100	0.00%	0.845
Mucosal RSV vaccines	Placebo	Cough	5	1.86 (0.89, 3.89)	100	0.00%	0.751
Mucosal RSV vaccines	Placebo	Rhinorrhea	2	1.79 (1.00, 3.20)	100	0.00%	0.381
Mucosal tuberculosis vaccines	Injectable tuberculosis vaccines	Fever	2	1.63 (0.08, 32.05)	100	49.90%	0.158
Mucosal tuberculosis vaccines	Injectable tuberculosis vaccines	Myalgia	3	0.75 (0.15, 3.85)	100	47.30%	0.15
Mucosal tuberculosis vaccines	Injectable tuberculosis vaccines	Sore throat	2	1.01 (0.46, 2.22)	100	0.00%	0.532
Mucosal tuberculosis vaccines	Injectable tuberculosis vaccines	Headache	3	1.10 (0.69, 1.75)	100	0.00%	0.485
Mucosal pertussis vaccines	Placebo	Cough	2	0.75 (0.16, 3.64)	100	0.00%	0.55
Mucosal pertussis vaccines	Placebo	Headache	2	1.17 (0.64, 2.15)	100	0.00%	0.631
Mucosal pertussis vaccines	Placebo	Nasal congestion	2	2.30 (0.37, 14.38)	100	0.00%	0.837
Mucosal pertussis vaccines	Placebo	Rhinorrhea	2	1.31 (0.59, 2.92)	100	0.00%	0.742

## Data Availability

All data supporting reported results are available within the manuscript and the Supplementary Materials.

## Supplementary Materials

The following supporting information can be downloaded at: https://www.mdpi.com/article/10.3390/vaccines13080825/s1, Figure S1: Quality assessment of the included randomized controlled trials; Figure S2: Quality assessment of the included non-randomized controlled trials; Figure S3: The seroconversion rate of sIgA for mucosal influenza vaccines (A/H1N1); Figure S4: The seroconversion rate of sIgA for mucosal influenza vaccines (A/H3N2); Figure S5: The seroconversion rate of sIgA for mucosal influenza vaccines (B); Figure S6: The pooled seroconversion rate of mucosal RSV vaccines; Figure S7: The pooled seroconversion rate of mucosal pertussis vaccines; Figure S8: The sensitivity analysis for immunogenicity of mucosal COVID-19 vaccines (nAb-GMT, Wide-type); Figure S9: The sensitivity analysis for immunogenicity of mucosal COVID-19 vaccines (nAb-GMT, Omicron); Figure S10: The sensitivity analysis for immunogenicity of mucosal COVID-19 vaccines (bAb-GMT of sIgA); Figure S11. The sensitivity analysis for immunogenicity of mucosal influenza vaccines (RR of seroconversion rate, A/H1N1); Figure S12: The sensitivity analysis for immunogenicity of mucosal influenza vaccines (RR of seroconversion rate, A/H2N3); Figure S13: The sensitivity analysis for immunogenicity of mucosal influenza vaccines (RR of seroconversion rate, B); Figure S14: The sensitivity analysis for immunogenicity of mucosal influenza vaccines (RR for seroconversion rate of sIgA, A/H1N1); Figure S15: The sensitivity analysis for immunogenicity of mucosal influenza vaccines (RR for seroconversion rate of sIgA, A/H3N2). Figure S16: The sensitivity analysis for immunogenicity of mucosal influenza vaccines (RR for seroconversion rate of sIgA, B); Figure S17: The sensitivity analysis for immunogenicity of mucosal RSV vaccines (pooled seroconversion rate); Figure S18. The sensitivity analysis for immunogenicity of mucosal pertussis vaccines (pooled seroconversion rate); Figure S19: The sensitivity analysis for protective efficacy of mucosal COVID-19 vaccines (VE); Figure S20: The sensitivity analysis for protective efficacy of mucosal influenza vaccines (VE); Figure S21. The sensitivity analysis for safety of mucosal influenza vaccines (cough). Figure S22: The sensitivity analysis for safety of mucosal influenza vaccines (headache); Figure S23. The sensitivity analysis for safety of mucosal influenza vaccines (nasal congestion); Figure S24. The sensitivity analysis for safety of mucosal influenza vaccines (rhinorrhea); Figure S25: The sensitivity analysis for safety of mucosal influenza vaccines (sore throat). Table S1: PRISMA 2020 Checklist; Table S2: Search strategy by Pubmed and The Cochrane Central Register of Controlled Trials. Table S3: PICOS; Table S4: Quality assessment of randomized controlled clinical trials using the Cochrane risk of bias tool; Table S5: Quality assessment of non-randomized controlled clinical trials using the ROBINS-I tool; Table S6: Quality assessment of cohort studies using the Newcastle-Ottawa quality assessment scale tool; Table S7: The result data of Egger’s test (publication bias).
