# Supplementary material for: Immunogenicity, Safety, and Protective Efficacy of Mucosal Vaccines Against Respiratory Infectious Diseases: A Systematic Review and Meta-Analysis
Source: Vaccines (Basel). 2025 Jul 31;13(8):825. doi: 10.3390/vaccines13080825 (PMC12389829; doi:10.3390/vaccines13080825)

Supplementary Materials Table S1. PRISMA 2020 Checklist.

Section and Topic	Item #	Checklist item	Location where item is reported
**TITLE**			
Title	1	Identify the report as a systematic review.	Pg. 1
**ABSTRACT**			
Abstract	2	See the PRISMA 2020 for Abstracts checklist.	Pg. 1-2
**INTRODUCTION**			
Rationale	3	Describe the rationale for the review in the context of existing knowledge.	Pg. 3
Objectives	4	Provide an explicit statement of the objective(s) or question(s) the review addresses.	Pg. 4
**METHODS**			
Eligibility criteria	5	Specify the inclusion and exclusion criteria for the review and how studies were grouped for the syntheses.	Pg. 5
Information sources	6	Specify all databases, registers, websites, organizations, reference lists and other sources searched or consulted to identify studies.Specify the date when each source was last searched or consulted.	Pg. 5
Search strategy	7	Present the full search strategies for all databases, registers and websites, including any filters and limits used.	Table S2

Selection process	8	Specify the methods used to decide whether a study met the inclusion criteria of the review, including how many reviewers screened eachrecord and each report retrieved, whether they worked independently, and if applicable, details of automation tools used in the process.	Pg. 5
Data collection process	9	Specify the methods used to collect data from reports, including how many reviewers collected data from each report, whether they workedindependently, any processes for obtaining or confirming data from study investigators, and if applicable, details of automation tools used inthe process.	Pg. 5
Data items	10a	List and define all outcomes for which data were sought. Specify whether all results that were compatible with each outcome domain ineach study were sought (e.g. for all measures, time points, analyses), and if not, the methods used to decide which results to collect.	Pg. 5
	10b	List and define all other variables for which data were sought (e.g. participant and intervention characteristics, funding sources). Describeany assumptions made about any missing or unclear information.	Table S1
Study risk of bias assessment	11	Specify the methods used to assess risk of bias in the included studies, including details of the tool(s) used, how many reviewers assessedeach study and whether they worked independently, and if applicable, details of automation tools used in the process.	Pg. 5
Effect measures	12	Specify for each outcome the effect measure(s) (e.g. risk ratio, mean difference) used in the synthesis or presentation of results.	Pg. 6
Synthesis methods	13a	Describe the processes used to decide which studies were eligible for each synthesis (e.g. tabulating the study intervention characteristicsand comparing against the planned groups for each synthesis (item #5)).	Pg. 6
	13b	Describe any methods required to prepare the data for presentation or synthesis, such as handling of missing summary statistics, or dataconversions.	Pg. 6
	13c	Describe any methods used to tabulate or visually display results of individual studies and syntheses.	Pg. 6
	13d	Describe any methods used to synthesize results and provide a rationale for the choice(s). If meta-analysis was performed, describe themodel(s), method(s) to identify the presence and extent of statistical heterogeneity, and software package(s) used.	Pg. 6

Section and Topic	Item #	Checklist item	Location where item is reported
	13e	Describe any methods used to explore possible causes of heterogeneity among study results (e.g. subgroup analysis, meta-regression).	Pg. 6
	13f	Describe any sensitivity analyses conducted to assess robustness of the synthesized results.	Pg. 6
Reporting bias assessment	14	Describe any methods used to assess risk of bias due to missing results in a synthesis (arising from reporting biases).	Pg. 6
Certainty assessment	15	Describe any methods used to assess certainty (or confidence) in the body of evidence for an outcome.	None
**RESULTS**			
Study selection	16a	Describe the results of the search and selection process, from the number of records identified in the search to the number of studiesincluded in the review, ideally using a flow diagram.	Figure S1
	16b	Cite studies that might appear to meet the inclusion criteria, but which were excluded, and explain why they were excluded.	None
Study characteristics	17	Cite each included study and present its characteristics.	Table 1
Risk of bias in studies	18	Present assessments of risk of bias for each included study.	Table S4-6
Results of individual studies	19	For all outcomes, present, for each study: (a) summary statistics for each group (where appropriate) and (b) an effect estimate and itsprecision (e.g. confidence/credible interval), ideally using structured tables or plots.	Figure 2-4; Figure S1-S8

Results of syntheses	20a	For each synthesis, briefly summarise the characteristics and risk of bias among contributing studies.	Pg. 6-7
	20b	Present results of all statistical syntheses conducted. If meta-analysis was done, present for each the summary estimate and its precision(e.g. confidence/credible interval) and measures of statistical heterogeneity. If comparing groups, describe the direction of the effect.	Pg. 28-37
	20c	Present results of all investigations of possible causes of heterogeneity among study results.	Pg. 37
	20d	Present results of all sensitivity analyses conducted to assess the robustness of the synthesized results.	Pg. 37
Reporting biases	21	Present assessments of risk of bias due to missing results (arising from reporting biases) for each synthesis assessed.	Pg. 37
Certainty of evidence	22	Present assessments of certainty (or confidence) in the body of evidence for each outcome assessed.	Pg. 37
**DISCUSSION**			
Discussion	23a	Provide a general interpretation of the results in the context of other evidence.	Pg. 37-38
	23b	Discuss any limitations of the evidence included in the review.	Pg. 38
	23c	Discuss any limitations of the review processes used.	Pg. 39
	23d	Discuss implications of the results for practice, policy, and future research.	Pg. 39
**OTHER INFORMATION**			
Registration and protocol	24a	Provide registration information for the review, including register name and registration number, or state that the review was not registered.	Pg. 4
	24b	Indicate where the review protocol can be accessed, or state that a protocol was not prepared.	Pg. 4

	24c	Describe and explain any amendments to information provided at registration or in the protocol.	None
Support	25	Describe sources of financial or non-financial support for the review, and the role of the funders or sponsors in the review.	Pg. 40
Competing interests	26	Declare any competing interests of review authors.	Pg. 40
Availability of data, code and other materials	27	Report which of the following are publicly available and where they can be found: template data collection forms; data extracted fromincluded studies; data used for all analyses; analytic code; any other materials used in the review.	Table 1

From: Page MJ, McKenzie JE, Bossuyt PM, et al. The PRISMA 2020 statement: an updated guideline for reporting systematic reviews. *BMJ* 2021;372:n71. doi:10.1136/bmj.n71

For more information, visit: **www.prisma-statement.org**.

Supplementary Materials Table S2. Search strategy by Pubmed and The Cochrane Central Register of Controlled Trials.

Database	Result	Search strings
Pubmed	15356 studies	#1 vaccines [MeSH] OR vaccination [Mesh] OR immunization [MeSH] OR vaccine*[Title/Abstract] OR immunize*[Title/Abstract]<br><br>#2 "inhalation*"[Title/Abstract] OR "inhale*"[Title/Abstract] OR "Inhalation"[MeSH] OR "administration, inhalation"[MeSH Terms] OR "Administration, Intranasal"[MeSH] OR "aerosolize*"[Title/Abstract]<br><br>#3 Administration, Intranasal [MeSH] OR Nasal Sprays [MeSH] OR Sprays, Nasal [Title/Abstract] OR Nasal Spray [Title/Abstract] OR Spray, Nasal [Title/Abstract] OR Nasal Aerosol [Title/Abstract] OR Aerosol, Nasal [Title/Abstract] OR Nasal Mist [Title/Abstract] OR Mist, Nasal [Title/Abstract]
The Cochrane Central Register of Controlled Trials	1928 studies	#1 MeSH descriptor: [Vaccines] explode all trees<br><br>#2 (vaccine* or immunize* or immunize* or inoculation or booster or vaccine booster or booster shot) :ti,ab,kw (Word variations have been searched)<br><br>#3 #1 or #2<br><br>#4 MeSH descriptor: [Inhalation] explode all trees<br><br>#5 MeSH descriptor: [Administration, Inhalation] explode all trees<br><br>#6 inhalation* OR inhale* OR Inhalation OR administration, inhalation OR aerosolize* OR nebulize* OR Intranasal<br><br>#7 #4 or #5 or #6<br><br>#8 MeSH descriptor: [Nasal Sprays] explode all trees<br><br>#9 Spray OR Nasal OR Nasal Aerosol OR Nasal Spray OR Aerosol OR Nasal Sprays OR Nasal OR Mist, Nasal OR Nasal Mist<br><br>#10 #8 or #9<br><br>#11 #3 and #7 and #10

Supplementary Materials Table S3. PICOS.

Parameter	Description
Population	Healthy population
Intervention	Mucosal vaccines (inhalation and intranasal spray)
Comparison	Traditional intramuscular or intradermal vaccines or placebo
Outcome	Immunogenicity, safety, and protective efficacy of mucosal vaccines
Study design	Randomized control trails and observational clinical trials and non- randomized control trails

Supplementary Materials Table S4. Quality assessment of randomized controlled clinical trials using the Cochrane risk of bias tool.

Study	Risk levels	Random sequence generation (selection bias)	Allocation concealment (selection bias)	Blinding of participants and personnel (performance bias)	Blinding of outcome assessment (detection bias)	Incomplete outcome data (attrition bias)	Selective reporting (reporting bias)	Other bias
Satti (2024)	low	low	low	low	low	low	low	low
Satti (2014)	low	low	low	low	low	low	low	low
Audran (2024)	low	low	low	low	low	low	low	low
Thomas (2019)	high	low	low	high	low	low	low	low
Marshall (2020)	low	low	low	low	low	low	low	low
Creech (2022)	high	low	low	high	low	Some concerns	Some concerns	low
Keech (2023)	low	low	low	low	low	low	low	low
Thorstensson (2014)	high	high	low	low	high	low	low	low
Green (2019)	high	high	high	high	low	low	low	low
Verdijk (2020)	low	low	low	low	low	low	low	low
Karron (2023)	low	Some concerns	low	low	low	low	low	low

Cunningham (2022)	Some concerns	Some concerns	Some concerns	Some concerns	Some concerns	low	low	low
Williams (2023)	high	low	low	high	low	low	low	low
Voorthuizen (1981)	low	low	low	low	low	low	low	low
Vesikari (2008)	low	low	low	low	low	low	low	low
Plas (2024)	low	low	low	low	low	low	low	low
Treanor (1999)	low	Some concerns	low	low	low	low	low	low
Tasker (2021)	low	low	low	low	low	low	low	low
Sambhara (2024)	low	low	low	low	low	low	low	low
Rudenko (2015)	low	low	low	low	low	low	low	low
Rudenko (2014)	low	low	low	low	low	low	low	low
Rudenko (2016)	low	low	low	low	low	low	low	low
Pitisuttithum (2017)	low	low	low	low	low	low	low	low
Phonrat(2013)	low	Some concerns	low	low	low	low	low	low
Pan (2020)	low	Some concerns	low	low	low	low	low	low
Nichol (1999)	low	low	low	low	low	low	low	low

Nakayam (2024)	low	low	low	low	low	low	low	low
Mallory (2010)	low	low	low	low	low	low	low	low
Li (2022)	low	low	low	low	low	low	low	low
Krishnan (2021)	low	low	low	low	low	Some concerns	low	low
Kiseleva (2020)	high	Some concerns	low	low	low	high	low	low
Halperin (2005)	high	low	low	high	low	Some concerns	low	low
Forrest (2011)	high	low	low	high	high	Some concerns	low	low
Block (2007)	low	low	low	low	low	low	low	low
Ambrose (2013)	high	low	high	high	high	low	low	low
Ai (2024)	low	Some concerns	low	low	low	low	low	low
Gruber (1993)	high	low	Some concerns	high	high	high	Some concerns	Some concerns
Zhu (2023)	Some concerns	low	low	low	Some concerns	low	low	low
Zhu (2022)	low	low	low	low	low	low	low	low
Zhang (2023)	low	low	low	low	low	low	low	low
Xu (2024)	Low	Low	Low	Low	Low	Low	Low	Low

Wu (2021)	high	low	low	high	low	low	low	low
Tang (2023)	high	low	low	high	low	low	low	low
Singh (2023)	high	Low	Some concerns	high	high	low	low	low
Li (2022)	high	low	low	high	low	low	low	low
Li (2023)	high	low	low	high	low	low	low	low
Jin (2023)	high	Some concerns	Some concerns	high	high	low	low	low
Huang (2023)	high	low	low	high	low	low	low	low
Dodaran (2023)	low	low	low	low	low	low	low	low

The risk of bias for each domain can be categorized into three levels: "Low" (low risk of bias), "some concerns" (some concerns) and " high risk" (high risk of bias). If the risk of bias evaluation results of all areas is "Low", then the overall risk of bias is "Low"; if the risk of bias evaluation results of some areas is "some concerns", then the overall risk of bias is "Low". If the risk of bias evaluation result of some areas is "some concerns" and there is no area of "high risk", then the overall risk of bias is "some concerns"; as long as there is an area where the risk of bias evaluation result is "low", then the overall risk of bias is "low". As long as there is one area where the risk of bias evaluation result is "high risk", then the overall risk of bias is "high risk".

Supplementary Materials Table S5. Quality assessment of non-randomized controlled clinical trials using the ROBINS-I tool.

Study	Risk levels	Bias due to confoundin g	Bias in selection of participants into the study	Bias in classification of interventions	Bias due to deviations from intended interventions	Bias due to missing data	Bias in measurement of the outcome	Bias in selection of the reported results
Jeyanatha (2022)	low	low	low	low	low	low	low	low
Spearman (2023)	low	low	low	low	low	low	low	low
Tong (2023)	moderate	moderate	low	low	low	low	low	low
Speroni (2005)	moderate	moderate	moderate	low	low	moderate	low	low
Hammitt (2009)	moderate	moderate	low	low	moderate	low	low	moderate
Manenti (2017)	moderate	moderate	low	low	low	low	low	low
Zhong (2022)	moderate	moderate	moderate	low	moderate	low	Low	moderate
Zhang (2023)	moderate	low	moderate	low	low	low	low	low
Sun (2024)	moderate	moderate	low	low	low	moderate	low	low
Madhava (2022)	moderate	moderate	moderate	low	moderate	low	low	moderate
There are 5 levels of judgement: low risk of bias, moderate risk of bias, Serious risk of bias, Critical risk of bias, no information.								

Supplementary Materials Table S6. Quality assessment of cohort studies using the Newcastle-Ottawa quality assessment scale tool.

Author	Year	Selection				Comparability	Outcome			Total score	Risk level[#]
		Representativeness of the Exposed Cohort	Selection of the Non-Exposed Cohort	Ascertainment of Exposure	Demonstration That Outcome of Interest Was Not Present at Start of Study		Assessment of Outcome	Was Follow-Up Long Enough for Outcomes to Occur	Adequacy of Follow Up of Cohorts		
Wang	2009	1	1	1	0	2	0	1	1	7	low
Rigamonti	2024	1	0	1	1	2	0	1	1	7	low
Gasparini	2021	1	0	1	0	0	1	1	0	4	high
Wang	2023	1	1	1	1	2	1	1	1	9	low
Wang	2024	1	1	1	1	2	1	1	1	9	low
Mi	2024	1	1	1	1	2	1	1	1	9	low

Low (total score ⩾ 7), moderate (total score 5-6), and high (total score ⩽ 4) risk of bias.

Supplementary Materials Table S7. The result data of Egger's test (publication bias).

Vaccines	Outcome	Subtype	Number of studies	Coefficient	Standard error	T	P	lower 95% CI	Upper 95% CI
Mucosal COVID-19 vaccines	Immunogenicity(nAb-GMT)	Wide-type	7	3.28	1.74	1.89	0.12	-1.19	7.76
Mucosal COVID-19 vaccines	Immunogenicity(nAb-GMT)	Omicron	8	2.54	3.95	0.64	0.54	-7.13	12.21
Mucosal COVID-19 vaccines	Immunogenicity（sIgA bAb-GMT)	Wide-type	4	3.81	3.76	1.01	0.418	-12.38	20.00
Mucosal	Protective vaccines efficacy (RR)	/	4	7.59	4.02	1.89	0.20	-9.69	24.87

COVID-19 vaccines									
Mucosal influenza vaccines	Immunogenicity (seroconversion rates)	A/H1N1	13	4.64	2.01	2.31	0.07	0.21	9.06
Mucosal influenza vaccines	Immunogenicity (seroconversion rates)	A/H3N2	11	2.14	2.06	1.04	0.33	-2.51	6.79
Mucosal influenza vaccines	Immunogenicity (seroconversion rates)	B	9	-1.01	3.52	-0.29	0.78	-9.32	7.31
Mucosal influenza vaccines	Immunogenicity (seroconversion rates)	A/H1N1	5	0.27	7.76	0.03	0.98	-24.44	24.98
Mucosal influenza vaccines	Immunogenicity (seroconversion rates)	A/H3N2	4	4.76	3.97	1.20	0.35	-12.33	21.85
Mucosal influenza vaccines	Immunogenicity (seroconversion rates)	B	3	-5.43	1.66	-3.28	0.19	-26.49	25.63

Mucosal influenza vaccines	Protective vaccines efficacy (RR)	/	5	-3.35	5.47	-0.61	0.58	-20.77	14.07
Mucosal RSV vaccines	Immunogenicity (seroconversion rates)	/	4	-10.35	3.08	-3.36	0.08	-23.60	2.90
Mucosal pertussis vaccines	Immunogenicity (seroconversion rates)	/	4	-9.46	4.52	-2.10	0.17	-28.89	9.96
Mucosal influenza Vaccine	Safety (Cough)	/	8	-0.42	1.19	-0.36	0.73	-3.32	2.48
Mucosal influenza vaccines	Safety (Sore throat)	/	7	0.06	0.95	0.06	0.95	-2.39	2.51
Mucosal influenza vaccines	Safety (Headache)	/	9	-0.18	0.89	-0.2	0.85	-2.28	1.92
Mucosal influenza	Safety (Nasal congestion)	/	8	0.3	0.96	0.32	0.76	-2.03	2.64

vaccines									
Mucosal influenza vaccines	Safety (Rhinorrhea)	/	7	0.25	0.98	0.26	0.81	-2.27	2.77

Supplementary Materials Figure S1. Quality assessment of the included randomized controlled trials.

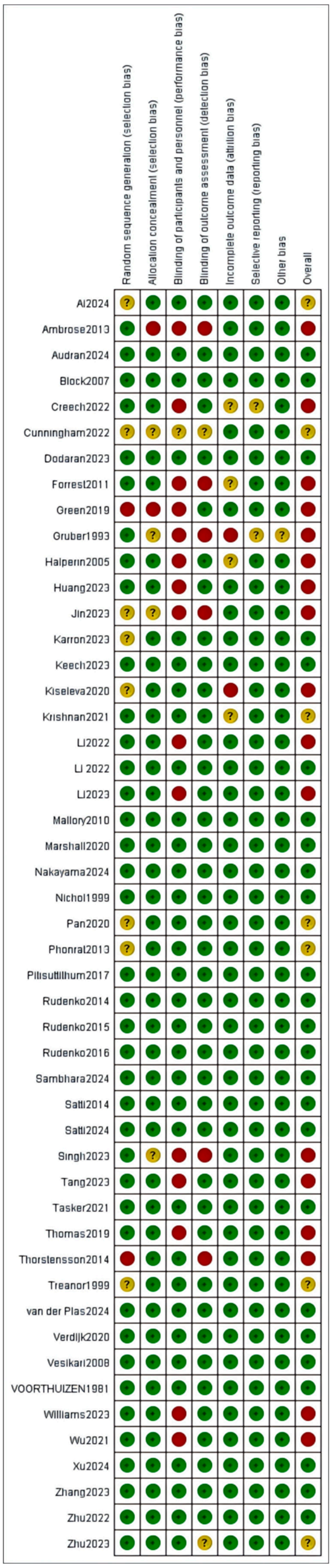

Supplementary Materials Figure S2. Quality assessment of the included non-randomized controlled trials.

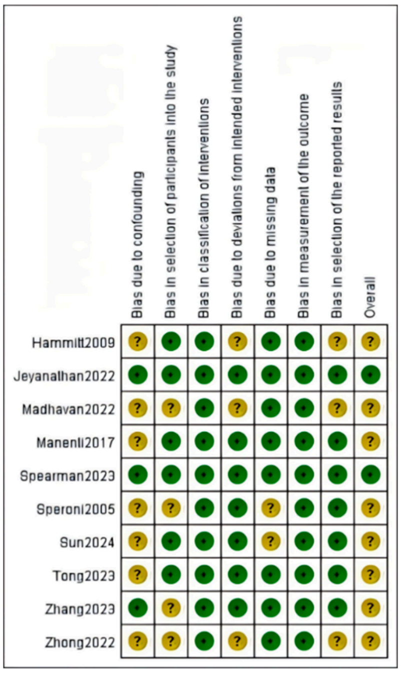

Supplementary Materials Figure S3. The seroconversion rate of sIgA for mucosal influenza vaccines (A/H1N1).

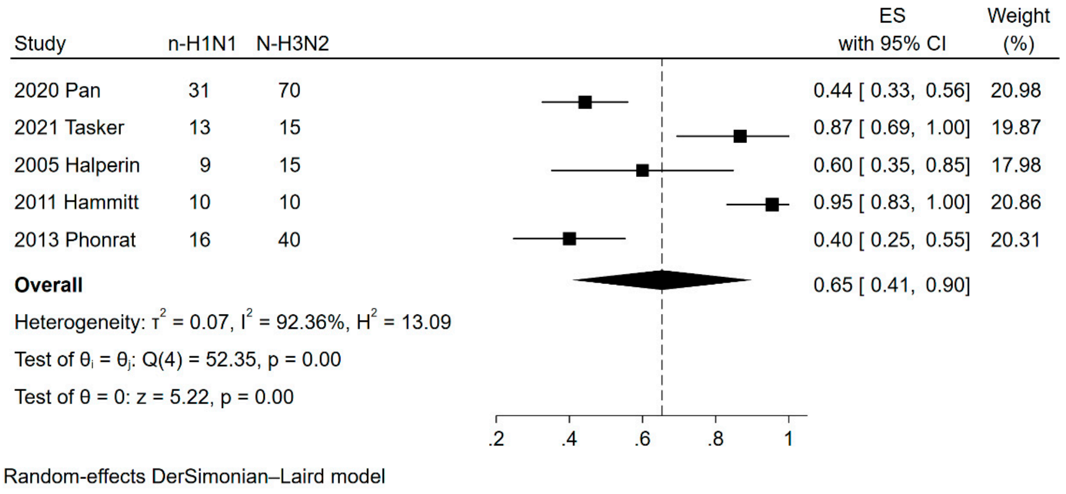

Supplementary Materials Figure S4. The seroconversion rate of sIgA for mucosal influenza vaccines (A/H3N2).

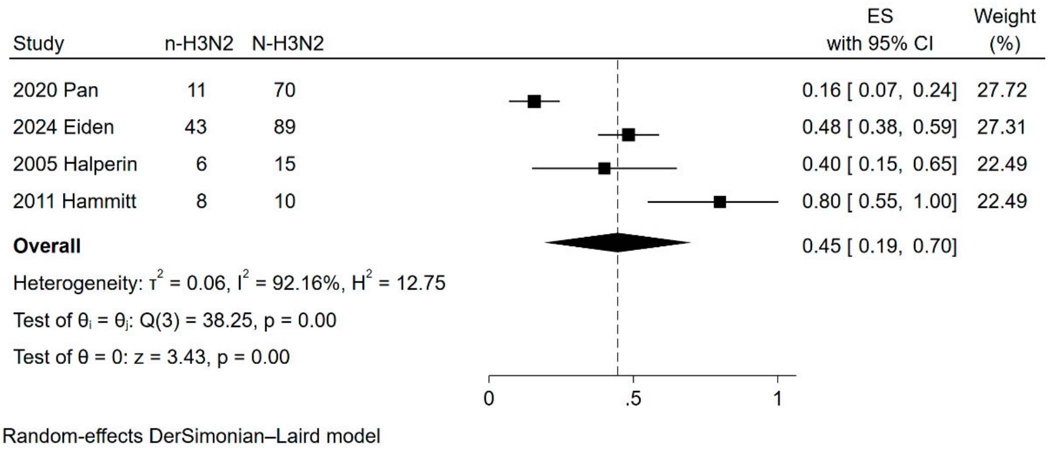

Supplementary Materials Figure S5. The seroconversion rate of sIgA for mucosal influenza vaccines (B).

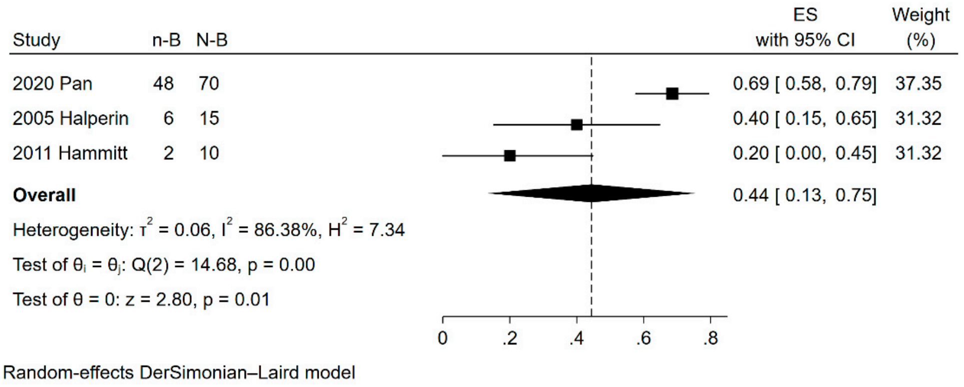

Supplementary Materials Figure S6. The pooled seroconversion rate of mucosal RSV vaccines.

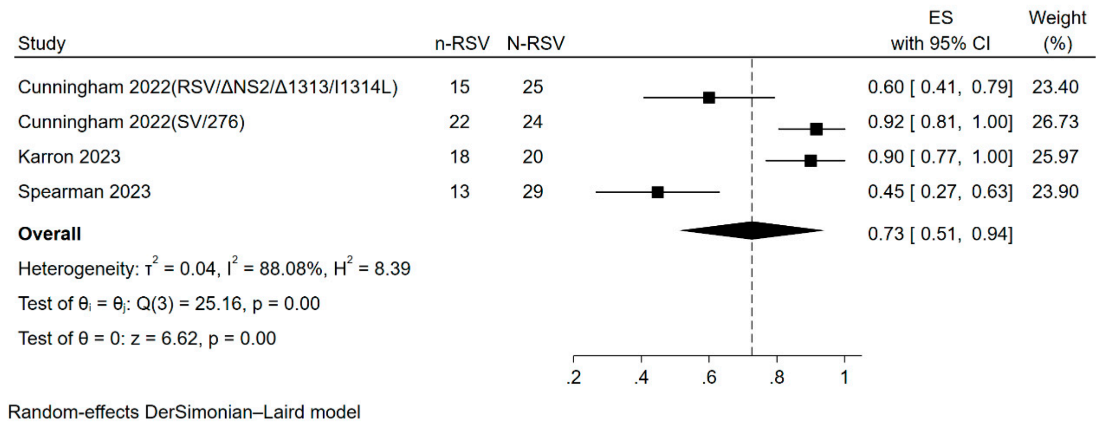

n-RSV: number of incidents; N-RSV: total number of participants.

Supplementary Materials Figure S7. The pooled seroconversion rate of mucosal pertussis vaccines.

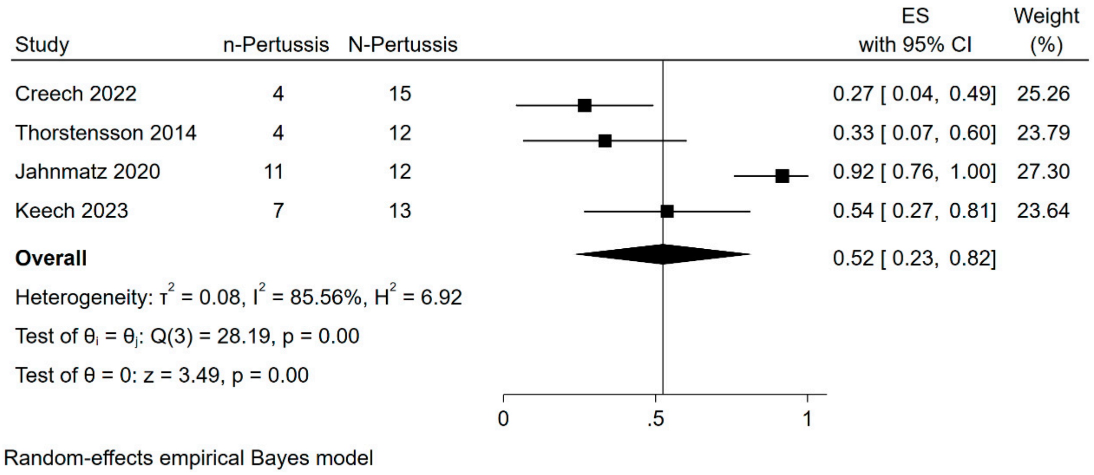

n-pertussis: number of incidents; N- pertussis: total number of participants.

Supplementary Materials Figure S8. The sensitivity analysis for immunogenicity of mucosal COVID-19 vaccines (nAb-GMT, Wide-type).

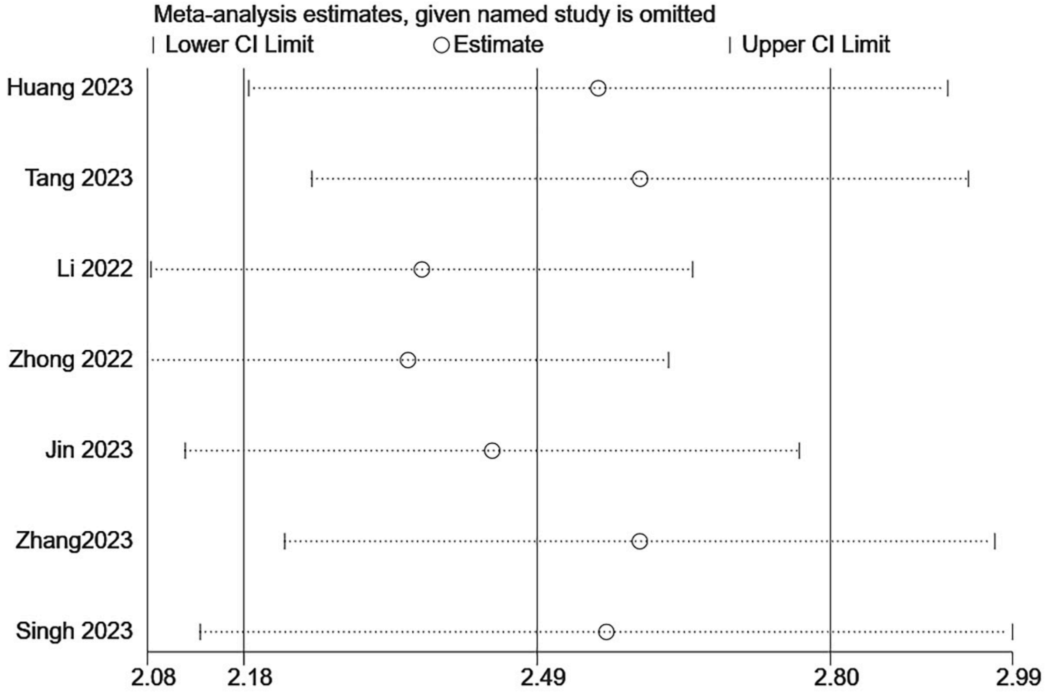

Supplementary Materials Figure S9. The sensitivity analysis for immunogenicity of mucosal COVID-19 vaccines (nAb-GMT, Omicron).

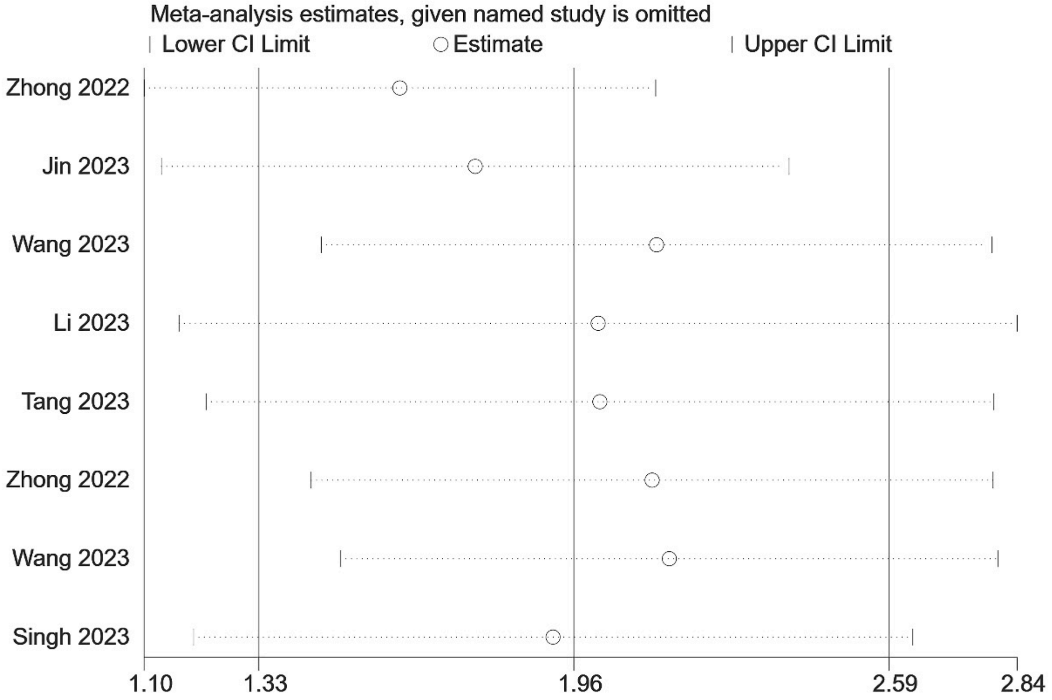

Supplementary Materials Figure S10. The sensitivity analysis for immunogenicity of mucosal COVID-19 vaccines (bAb-GMT of sIgA).

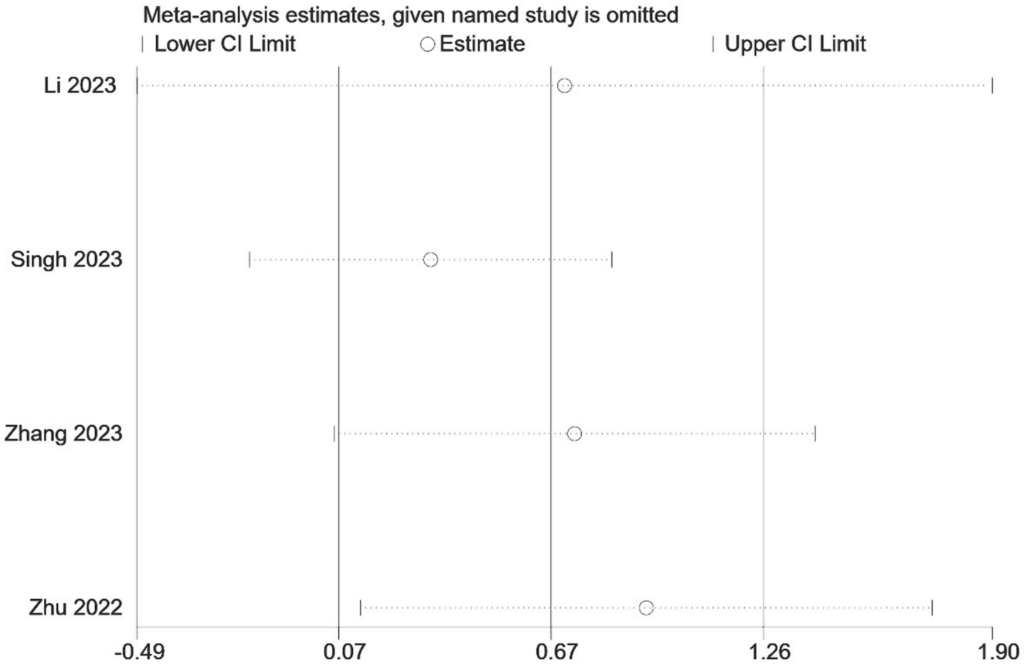

Supplementary Materials Figure S11. The sensitivity analysis for immunogenicity of mucosal influenza vaccines (RR of seroconversion rate, A/H1N1).

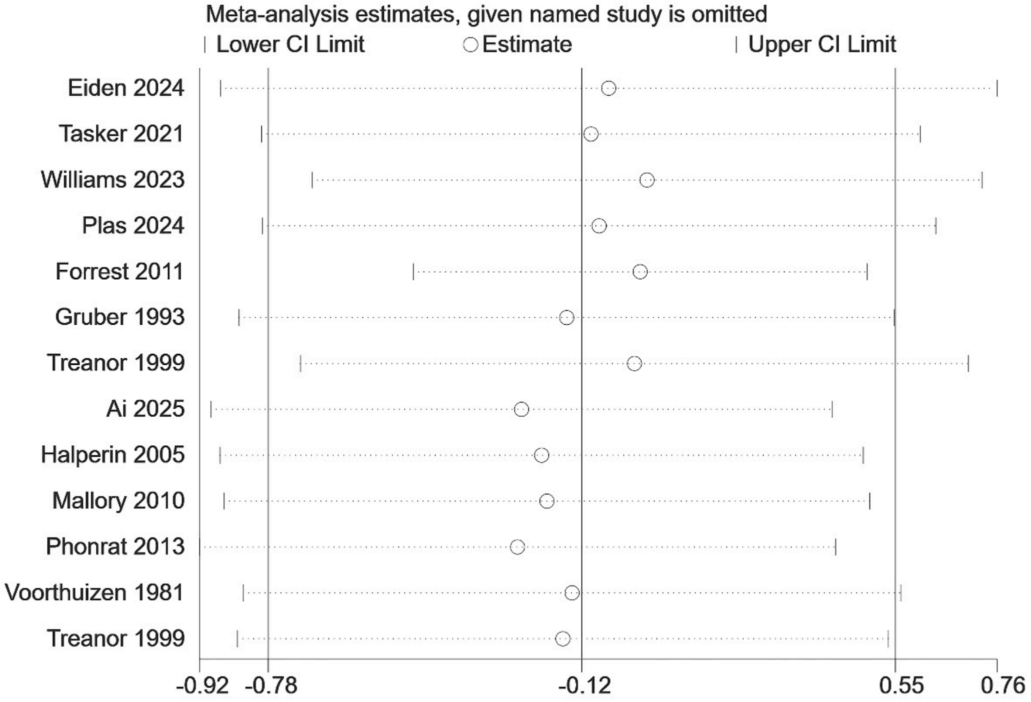

Supplementary Materials Figure S12. The sensitivity analysis for immunogenicity of mucosal influenza vaccines (RR of seroconversion rate, A/H2N3).

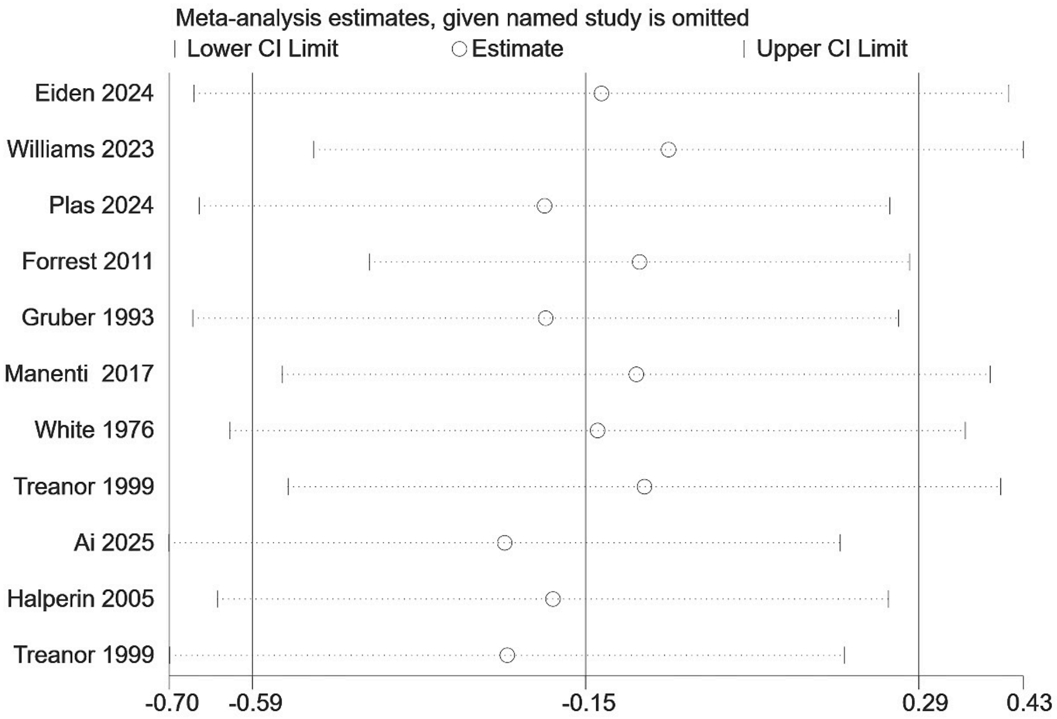

Supplementary Materials Figure S13. The sensitivity analysis for immunogenicity of mucosal influenza vaccines (RR of seroconversion rate, B).

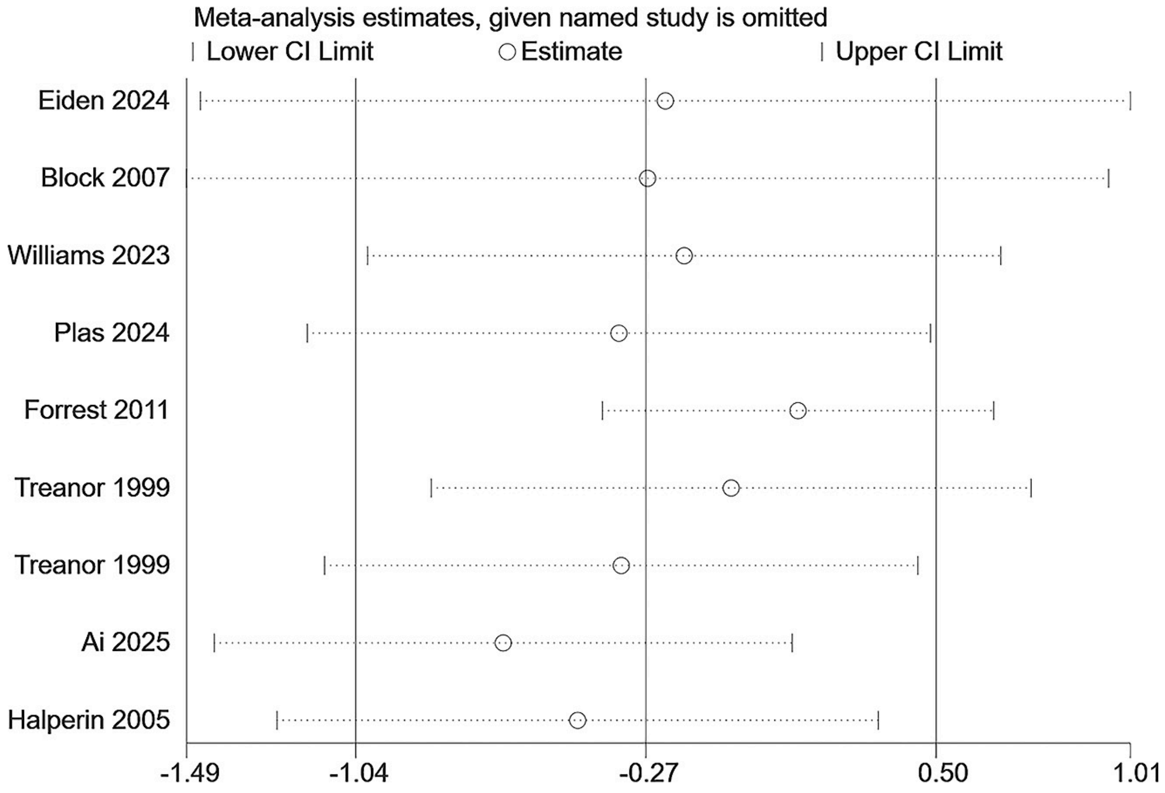

Supplementary Materials Figure S14. The sensitivity analysis for immunogenicity of mucosal influenza vaccines (RR for seroconversion rate of sIgA, A/H1N1).

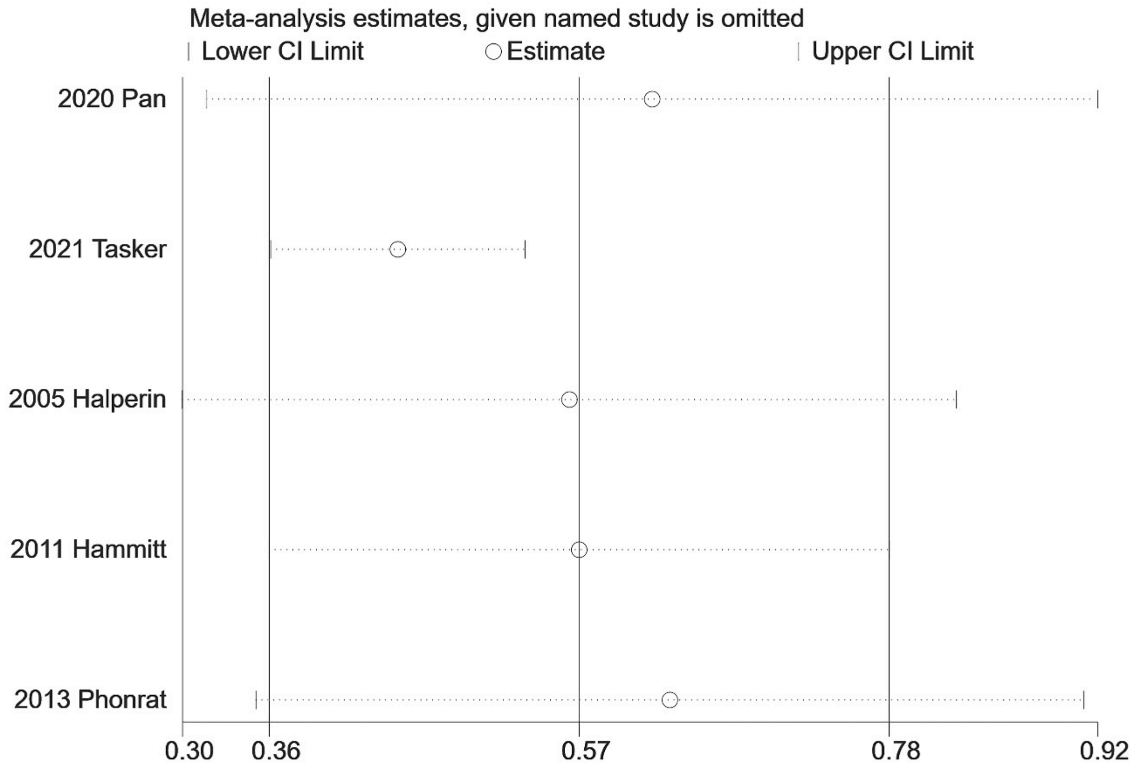

Supplementary Materials Figure S15. The sensitivity analysis for immunogenicity of mucosal influenza vaccines (RR for seroconversion rate of sIgA, A/H3N2).

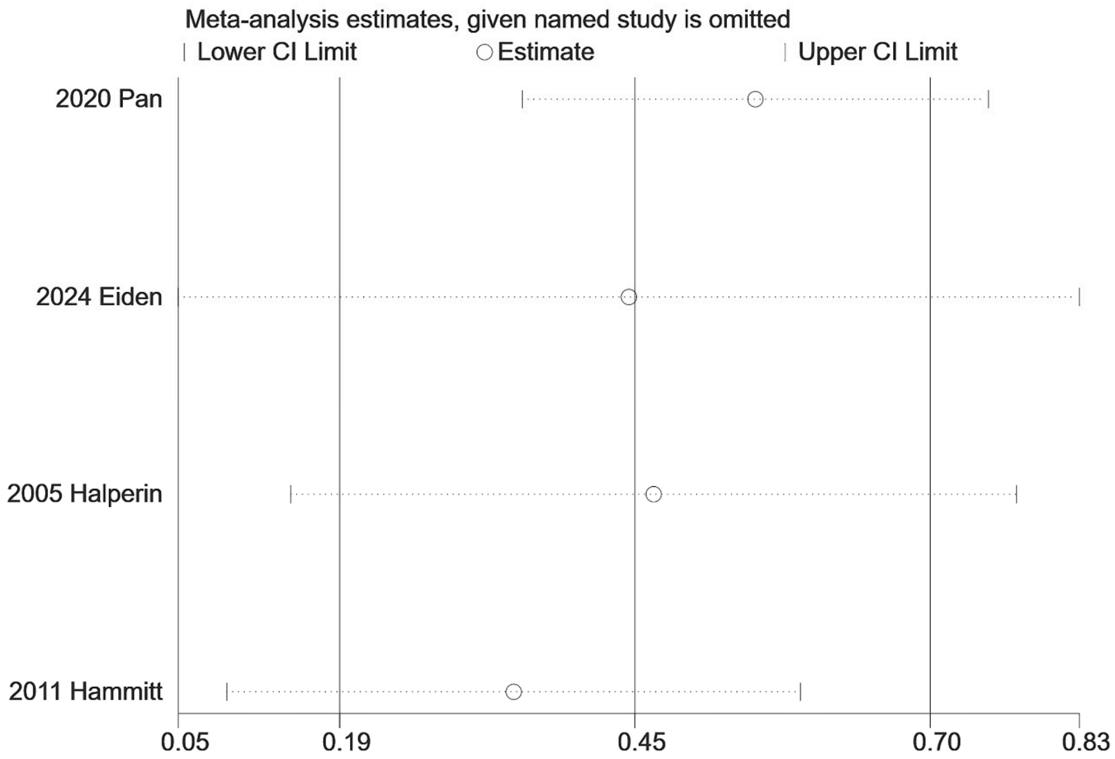

Supplementary Materials Figure S16. The sensitivity analysis for immunogenicity of mucosal influenza vaccines (RR for seroconversion rate of sIgA, B).

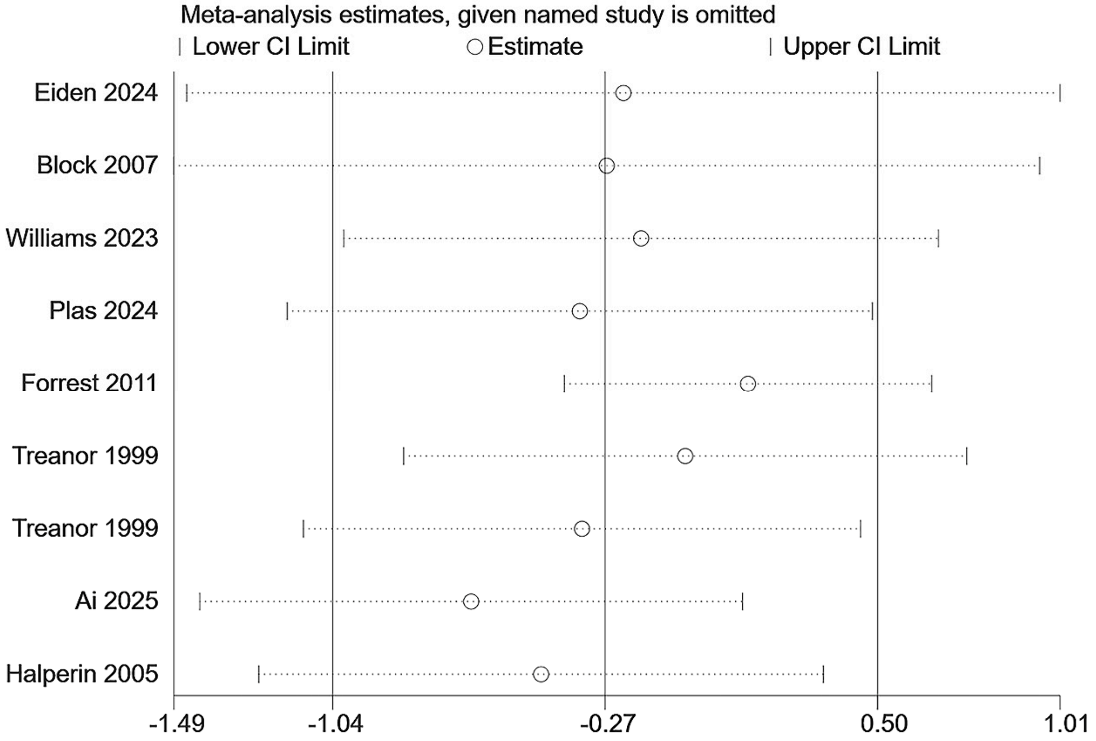

Supplementary Materials Figure S17. The sensitivity analysis for immunogenicity of mucosal RSV vaccines (pooled seroconversion rate).

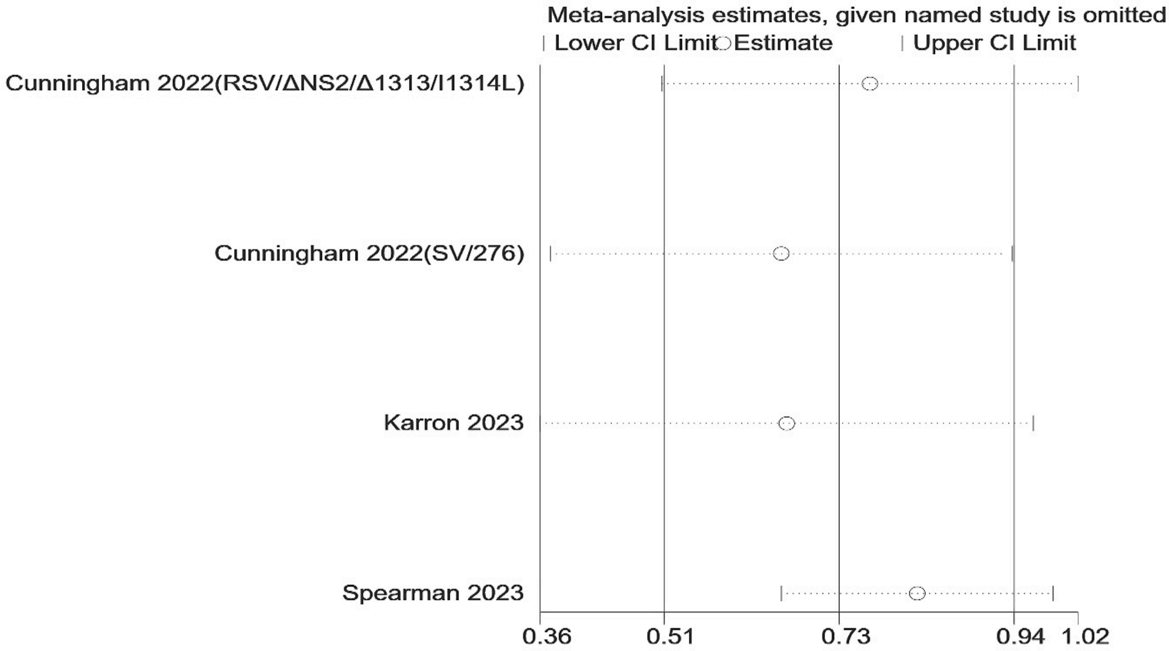

Supplementary Materials Figure S18. The sensitivity analysis for immunogenicity of mucosal pertussis vaccines (pooled seroconversion rate).

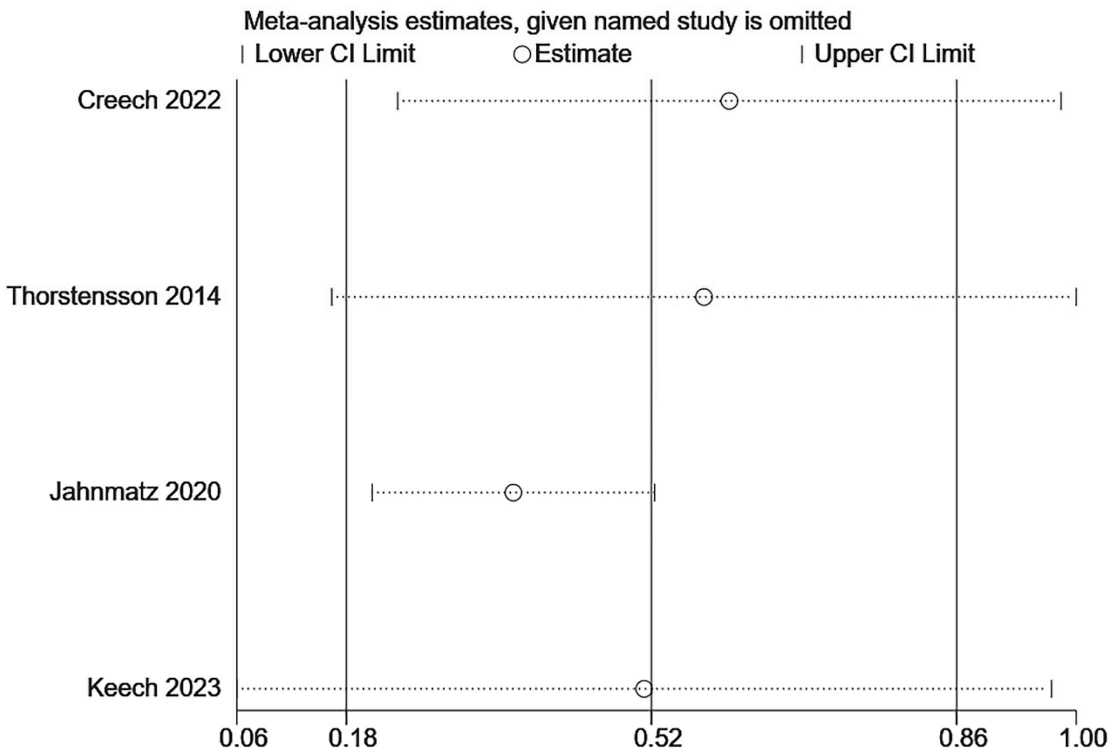

Supplementary Materials Figure S19. The sensitivity analysis for protective efficacy of mucosal COVID-19 vaccines (VE).

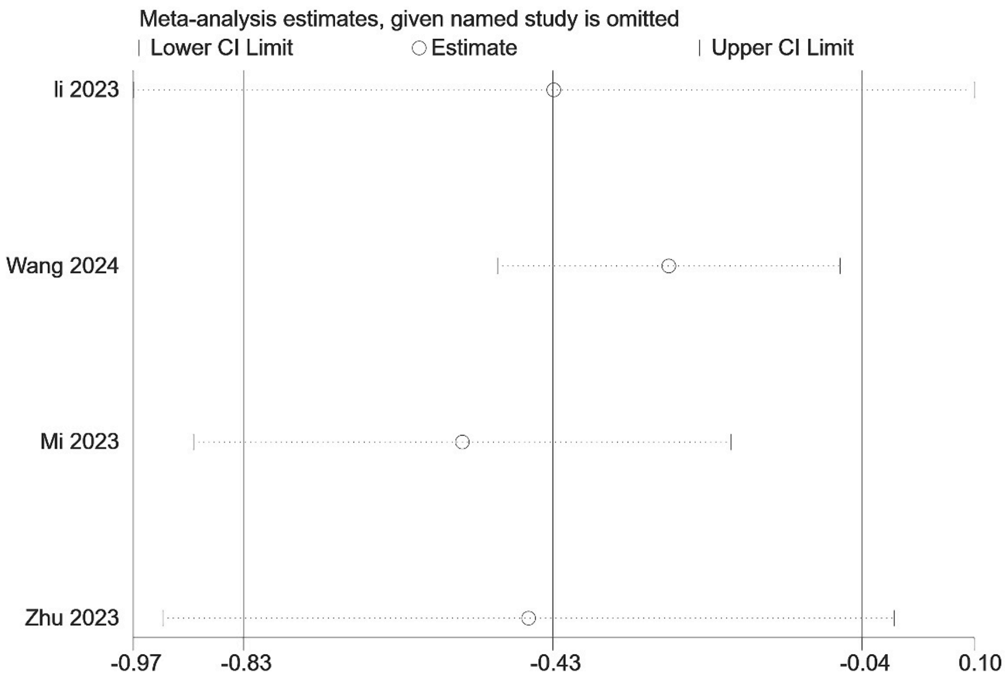

Supplementary Materials Figure S20. The sensitivity analysis for protective efficacy of mucosal influenza vaccines (VE).

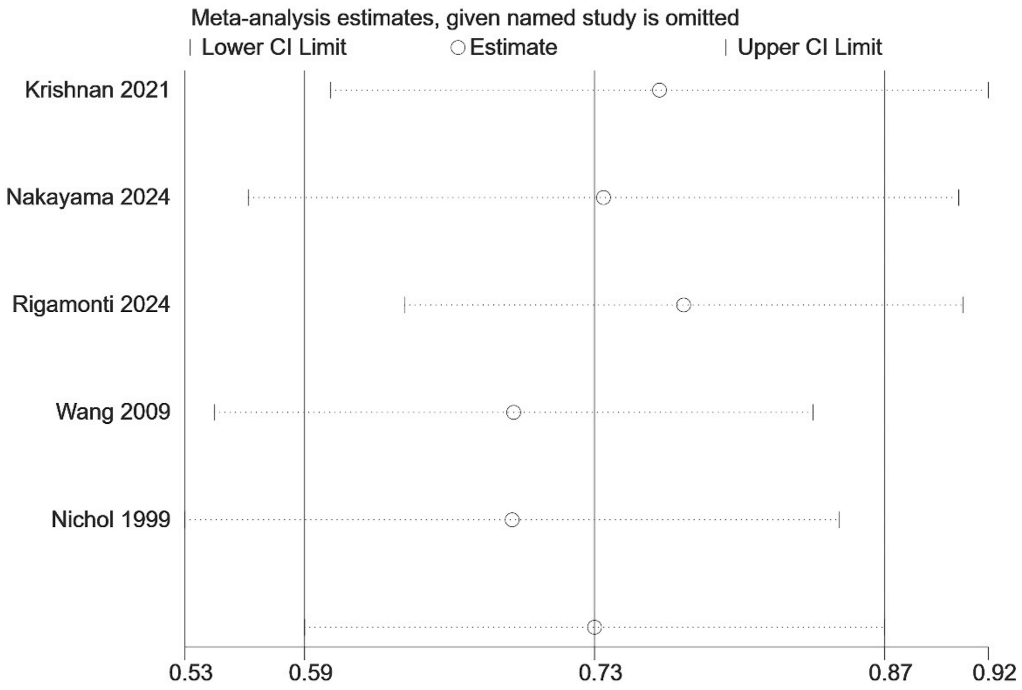

Supplementary Materials Figure S21. The sensitivity analysis for safety of mucosal influenza vaccines (cough).

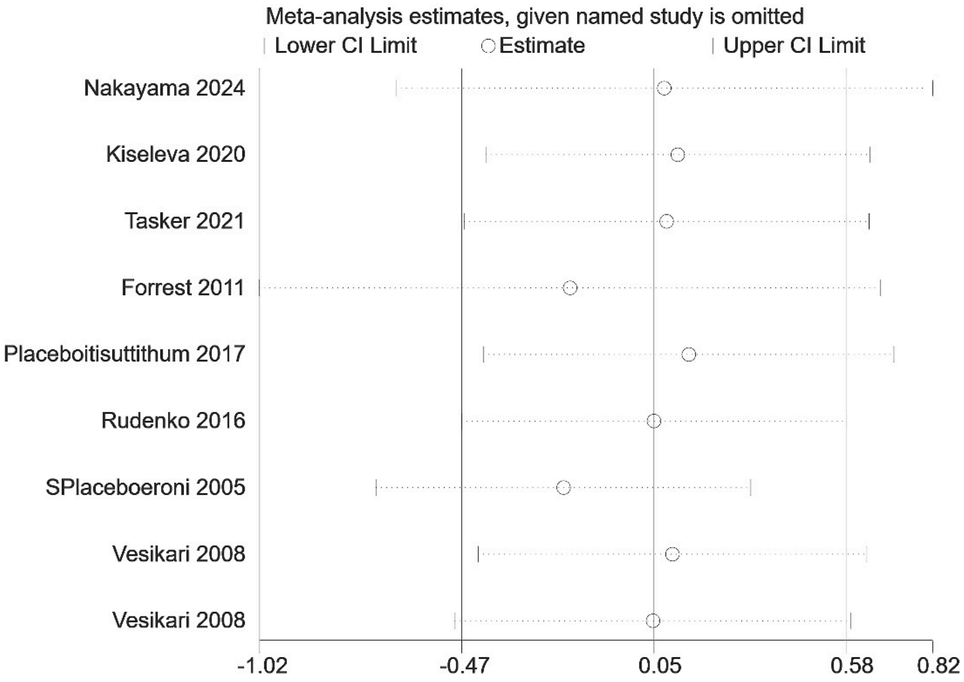

Supplementary Materials Figure S22. The sensitivity analysis for safety of mucosal influenza vaccines (headache).

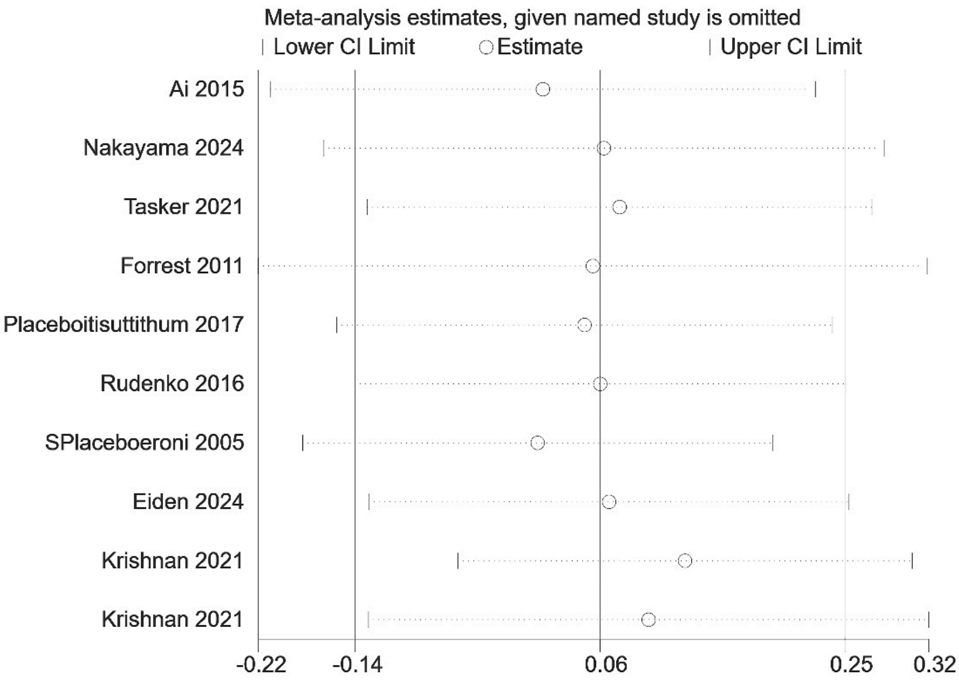

Supplementary Materials Figure S23. The sensitivity analysis for safety of mucosal influenza vaccines (nasal congestion).

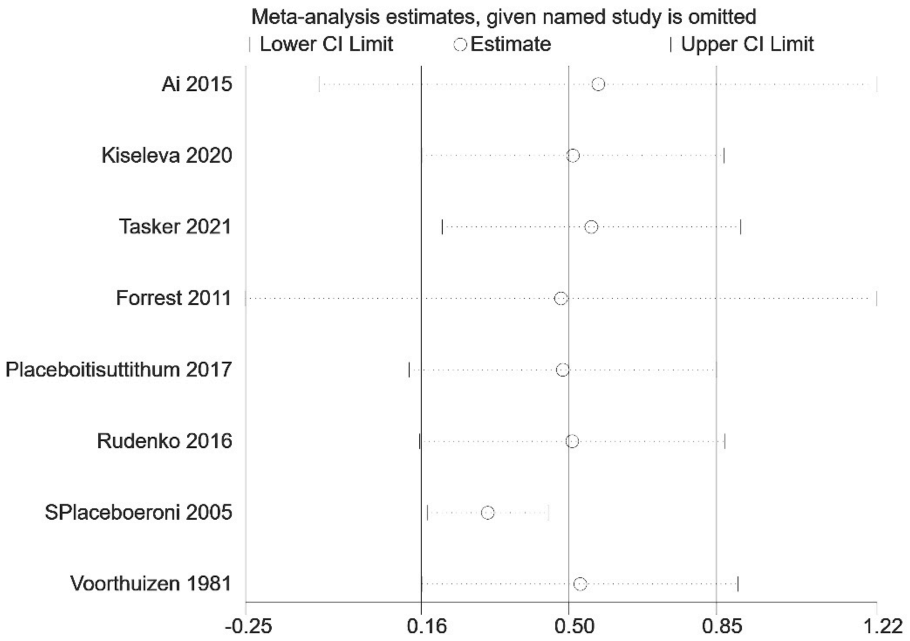

Supplementary Materials Figure S24. The sensitivity analysis for safety of mucosal influenza vaccines (rhinorrhea).

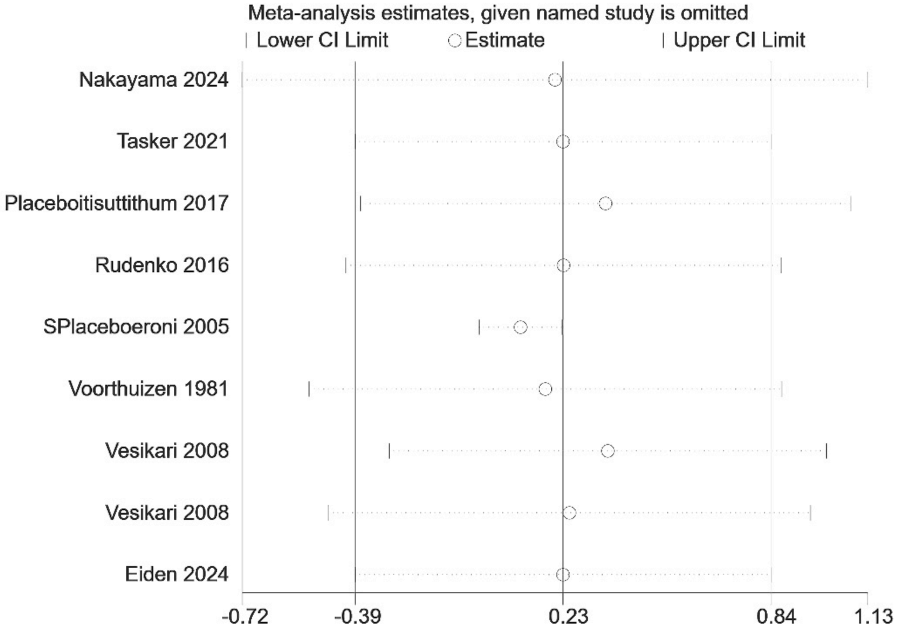

Supplementary Materials Figure S25. The sensitivity analysis for safety of mucosal influenza vaccines (sore throat).

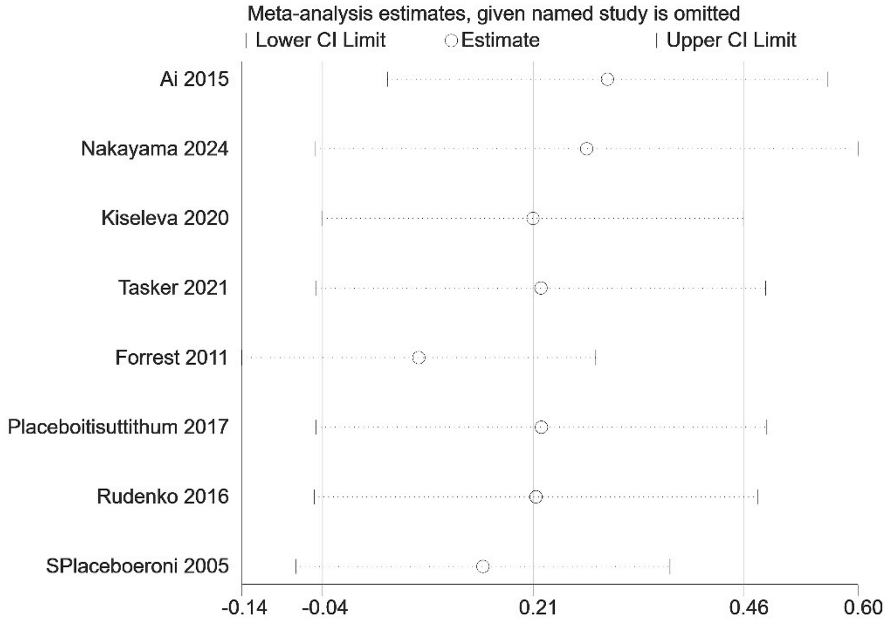

Supplement: Supplementary file 1 [file vaccines-13-00825-s001.zip › vaccines-3732593-supplementary.pdf]
